# The First Modern Human Dispersals across Africa

**DOI:** 10.1371/journal.pone.0080031

**Published:** 2013-11-13

**Authors:** Teresa Rito, Martin B. Richards, Verónica Fernandes, Farida Alshamali, Viktor Cerny, Luísa Pereira, Pedro Soares

**Affiliations:** 1 IPATIMUP (Instituto de Patologia e Imunologia Molecular da Universidade do Porto), Porto, Portugal; 2 School of Applied Sciences, University of Huddersfield, QueensGate, Huddersfield, United Kingdom; 3 Institute of Integrative and Comparative Biology, Faculty of Biological Sciences, University of Leeds, Leeds, United Kingdom; 4 Dubai Police GHQ - General Department of Forensic Sciences & Criminology, Dubai, United Arab Emirates; 5 Department of Anthropology and Human Genetics, Faculty of Science, Charles University, Prague, Czech Republic; 6 Institute for Advanced Study, Paris, France; 7 Faculdade de Medicina da Universidade do Porto, Porto, Portugal; Natural History Museum of Denmark, Denmark

## Abstract

The emergence of more refined chronologies for climate change and archaeology in prehistoric Africa, and for the evolution of human mitochondrial DNA (mtDNA), now make it feasible to test more sophisticated models of early modern human dispersals suggested by mtDNA distributions. Here we have generated 42 novel whole-mtDNA genomes belonging to haplogroup L0, the most divergent clade in the maternal line of descent, and analysed them alongside the growing database of African lineages belonging to L0’s sister clade, L1’6. We propose that the last common ancestor of modern human mtDNAs (carried by “mitochondrial Eve”) possibly arose in central Africa ~180 ka, at a time of low population size. By ~130 ka two distinct groups of anatomically modern humans co-existed in Africa: broadly, the ancestors of many modern-day Khoe and San populations in the south and a second central/eastern African group that includes the ancestors of most extant worldwide populations. Early modern human dispersals correlate with climate changes, particularly the tropical African “megadroughts” of MIS 5 (marine isotope stage 5, 135–75 ka) which paradoxically may have facilitated expansions in central and eastern Africa, ultimately triggering the dispersal out of Africa of people carrying haplogroup L3 ~60 ka. Two south to east migrations are discernible within haplogroup LO. One, between 120 and 75 ka, represents the first unambiguous long-range modern human dispersal detected by mtDNA and might have allowed the dispersal of several markers of modernity. A second one, within the last 20 ka signalled by L0d, may have been responsible for the spread of southern click-consonant languages to eastern Africa, contrary to the view that these eastern examples constitute relicts of an ancient, much wider distribution.

## Introduction

There is a broad consensus that Africa was the birthplace of *Homo sapiens* – and what have been referred to as “anatomically modern humans” (AMH). However, the question of where in Africa this genesis took place, and even whether such a question can be considered meaningful, remains highly controversial [1,2]. The evidence comes mainly from four disciplines: palaeoanthropology, genetics, archaeology and palaeoclimatology. 

1. Based on fossil evidence, eastern Africa has often been considered the most likely location for the emergence of AMH. The Omo 1 cranium found in south-western Ethiopia and dating to ~190–200 ka (thousand years ago) is the oldest known fossil agreed to display AMH features [3] and alongside crania from Herto (in northern Ethiopia), dating to ~154–160 ka [4,5], and remains from Sudan and Tanzania [6], provide the palaeoanthropological case for an eastern African origin. Other early modern *Homo sapiens* remains include the Jebel Irhoud fossils in Morocco, also dating to ~160 ka [7], albeit with wide confidence intervals and some disagreement about their status [2], and the Skhul/Qafzeh remains in Israel, usually dated to ~90–135 ka [8] although the dates for Skhul are much less certain than those from Qafzeh (~85–95 ka) [9]. The oldest agreed AMH fossil known in southern Africa is from the Klasies River caves and dates to ~65–105 ka [9], although again its status as AMH has been contested [10]; although there are more archaic remains (notably from Florisbad, South Africa) dating to 190–330 ka [9], indicating that a southern origin is a possibility. Thus the fossil record is extremely patchy, but tends to point to a northern rather than southern, and in particular an eastern African, origin for AMH.

2. Genetically, the picture is equally cloudy. Two studies based on genome-wide data, one mainly comprising STRs (short tandem repeats) and indels [11] and another comprising >580,000 SNPs (single-nucleotide polymorphisms) [12] both suggest a southern African origin. Several autosomal genomic studies have suggested an early divergence between southern Khoesan populations and the remaining African populations [13–15], which does not indicate a southern origin but illustrates the dichotomy between north and south in ancient African prehistory. An analysis of the tree of the human male-specific Y-chromosome (MSY) with improved resolution located the root in central/west Africa, albeit with few data as yet [16]. Very recently a more divergent lineage was identified in an African American and at low frequencies in central Africa [17]. Although the divergence time of this lineage (>330 ka) considerably predates the generally accepted age of AMH, and might even suggest introgression from archaic humans, it does lend further support to origin of the modern human male lineage in central Africa. Finally, the maternally-inherited mitochondrial DNA (mtDNA) has given an ambiguous signal. The tree splits into two main branches, L1’6 and L0 [18]. L1’6 shows a central/eastern African origin (or at least an origin in the northern part of the continent: there is no mtDNA record for North Africa as it was probably depopulated in MIS 4, 75–60 ka [6] based on the archaeological record, and only repopulated after 50 ka from Eurasia [19,20]), or it underwent a population replacement around this time. This lineage ultimately gave rise to haplogroup L3 in eastern Africa, which dispersed out of Africa ~60–70 ka [21–24]. However, L0 most likely has a southern African origin on the basis of its prevalence in indigenous Khoe (both herder and hunter-gatherer) and San (Ju- and Tuu-speaking “Bushman” hunter-gatherer) populations, as well as in exclusively southern African Bantu speakers – although one of the main subclades of L0 (L0a’b’f) may have an eastern African origin [21,25–29]. Thus from the time after the main African clades have evolved they can prove highly informative; but the final path back to the human mtDNA root remains mysterious. 

Furthermore, it is not clear that different systems are informing us about the same phenomena. “Modern” features in human anatomy, especially the domed cranial vault [30], appear ~150–200 ka, and predominate in Africa after 130 ka, but there is no indication in the fossil record of a speciation event at this time, and *Homo sapiens* emerges gradually from more archaic specimens in Africa over the preceding few hundred thousand years [2,6]. It is often assumed that the age of the Most Recent Common Ancestor (MRCA) of modern mtDNAs, “mitochondrial Eve”, points to the timing of the appearance of modern *Homo sapiens*, but this is unwarranted unless there was a speciation bottleneck, the evidence for which is weak [31]. The coalescence time of the MSY for a long time seemed to be somewhat lower, even after the discovery of A1b (now A0) lineages, dating to ~140 ka, by Cruciani and colleagues [16] However, the recent identification of the much deeper A00 lineages, combined with the slower mutation rate calibrated from complete-MSY data, now suggests an MSY MRCA of almost 350 ka [17]. 

Similarly, the autosomes coalesce on average much earlier than either of the uniparental marker systems, so once again, even assuming that the region of highest autosomal diversity is the region with the highest time depth, this region would probably predate the appearance of modern human anatomy by hundreds of thousands of years – even in the case of a strong bottleneck and a single place of origin. Thus the region of highest diversity may differ for different marker systems and, despite having been commonly used as such, is not necessarily informative about the place of speciation. 

In addition, genetic diversity measures such as linkage disequilibrium (LD) and *F*_*ST*,_ which have been used to establish probable geographic origin of AMH [12], are very sensitive to demographic influences. From previous results we know that northern (i.e. central, eastern and west African) populations have experienced episodes of heavy exchange and gene flow (e.g. expansions within haplogroup L3 after 60 ka [22]) that could may have increased their LD values. They have also undergone extensive back-flow from Eurasian populations (via North Africa or Arabia/East Africa) long after the initial dispersal out of Africa [20,32–35], which has reduced their differentiation from non-Africans. For example, in eastern Africa the mtDNA gene pool shows up to one quarter of lineages with a recent out-of-Africa origin. By comparison, for South African Khoesan, substantial admixture with other very distinct populations seems unlikely, apart from the very recent Bantu genetic input [36]. 

3. The question of “the origins of modern humans” is inevitably intertwined with behavioural, social and cultural changes, and not only anatomical or genetic change. A substantial increase in symbolic and technological complexity in the archaeological record has been associated with modern behaviour, but for a long time was linked with the Eurasian Upper Palaeolithic transition ~50 ka and the “human revolution” model [37]. However, discoveries in sub-Saharan Africa in recent years, most spectacularly at Blombos Cave in South Africa – including bone tools, blade and microlithic technology, use of pigment, art and decoration [38] and dating at least 30 ka older than the Upper Palaeolithic – challenged this view [39]. Personal ornaments, regarded as an expression of symbolism and one of the clearest archaeological marker of “modern human behaviour”, have been found at the 75 ka levels of Blombos cave [40]. Engraved ochre has been further recovered from 75–100 ka in Blombos cave [38] and from 85–100 ka at the Klasies River caves, also in South Africa [41]. Shell ornaments of the same tick genus (*Nassarius*) are found in quantity in Morocco at ~70–85 ka [42,43] and there is some evidence for their use for personal decoration in Israel and Algeria by ~100 ka [44]. Use of marine resources and pigments, whilst less clearly diagnostic for modern behaviour, have been suggested to occur as early as ~165 ka in South Africa [45]. Recently, sophisticated and enduring microlithic technology has been argued to have arisen by 71 ka in South Africa [46]. Although the emphasis has been on eastern and southern Africa, other regions have been less well studied or have had poorer environments for establishing a long-term archaeological record, for example the Central and West African rainforest areas, leading to biases in the picture. Absence of archaeological or paleontological data should, of course, always be interpreted cautiously.

The archaeological record has led to the suggestion that anatomical and behavioural modernity arose together gradually, in piecemeal fashion, rather than being separated by a gulf of more than a hundred thousand years [47]. Even so, this observation simply contextualises, rather than erasing, the evidence for a “creative explosion” after ~75 ka [1,48]. It may have been simply demography, in terms of population size and interconnectedness, that allowed social and cultural innovations to “take hold” and survive [49]. This would chime with the view that “modernity” might be best characterised in terms of social networks and the “release from proximity” facilitated by symbolic exchange and language [1,48]. 

4. The impact of climate on demography has inevitably also been central to the debate. The suggestion that tropical central Africa may have experienced a series of “megadroughts” throughout much of MIS 5, from ~135–75 ka, followed by moister conditions ~75–60 ka, has led to an interpretation of the expansion of haplogroup L3 ~60–70 ka both across and out of Africa as climate-driven [22,50]. The climatic evidence is far from clear and difficult to interpret [51], but a recent synthesis provides an extremely valuable summary with which to compare the archaeological and genetic evidence [52]. It seems likely that the climate pattern in eastern Africa was less severe than in the tropical zone, and became moist earlier, ~80 ka. Moreover, Blome et al. [52] have argued that, within tropical Africa, human populations were relatively buffered from the effects of climate change, so this region may have provided refugia during the glacial MIS 6 (190–135 ka) and MIS 5. The megadrought period may, paradoxically, have increased the suitability of central Africa for human occupation by breaking up the dense tropical rainforest into a more open wooded environment [52].

Despite huge advances in genomics in recent years, the uniparental genetic systems remain the clearest markers of past human dispersals, since they allow the reconstruction of dated individual migration results on the basis of parsimony [53]. From the perspective of mtDNA, haplogroup L0, which resulted from the most ancient split in the mtDNA phylogeny and is largely restricted to eastern and southern Africa, is at the heart of debates about the early expansions of modern humans. Although it is found in a minority of people alive today (~11% of the African database), phylogenetically L0 represents half of modern human mtDNA diversity [28]. Here, as well as new sequence data, we also apply the improved molecular clock of Soares et al. [53], which allows for the effects of purifying selection, enabling us to draw direct parallels with the archaeological and freshly interpreted palaeoclimate evidence that were previously obscure. 

We show that the L0 phylogeography implies a number of very early dispersals between eastern, central and southern Africa – the earliest detectable migrations involving modern humans to date – with the deepest geographically restricted branching in all three regions occurring at ~130 ka. This was the time of onset of MIS 5 and the expansion of mode 3 technological industries throughout Africa, when the archaeological visibility of *Homo sapiens* in the African landscape dramatically increased [6]. Subsequent dispersals from southern towards eastern Africa occurred ~100 ka, perhaps involving the spread of personal ornaments, and again much more recently at ~ 7.5 ka, the latter of which may have carried southern click-consonant languages into eastern Africa.

## Materials and Methods

We generated 42 new whole-mtDNA genomes belonging to haplogroup L0, as described previously [54]. We performed the sequencing on a 3100 DNA Analyzer (AB Applied Biosystems) and analysed the resulting sequences with SeqScape (AB Applied Biosystems) and BioEdit version 7.0.4.1 [55], checked by two independent investigators. The samples included 14 from Mozambique, four from São Tomé e Princípe, eight from Somalia, two Nubians from Sudan, two Turkana from Kenya, two Sudanese Arabs, two Daza from Chad, one Fulani from Zinder area (Niger), one Kanembou from Chad, one Hide, one Bulahay and one Kotoco from Cameroon [56] and two Oromo and one further Ethiopian. Figure S1 displays the geographic location of the new sample. The 42 new whole-mtDNA sequences have been deposited in GenBank (accession numbers KF672796-KF672837).

The work was approved by the Ethics Committee of the University of Porto (11/CEUP/2011). For the sampling, local authorisations from Health or Science Ministries were obtained. In most cases (as for example in Daza, Hide, Oromo, Ethiopia) it was necessary to recur to an interpreter and informed consent was given orally as almost no one could write or read, or expressed themselves in local dialects. We documented information about the family structure provided by the individual, in order to avoid including closely related people in the study. The ethics committee approved this procedure. 

We used published whole-mtDNA and HVS-I (hypervariable segment I) sequences for comparison. We collected and analysed a total of 1250 L0 HVS-I sequences from the literature from a total of 11,015 African individuals surveyed (Table S1). HVS-I sequences were classified into L0a, L0b, L0d, L0f and L0k haplogroups using diagnostic control-region mutations present on the respective branches of the mtDNA tree [18]. A broader geographic classification into eastern, central, southern, west and North Africa, and the Sahel belt, was performed as previously [22].

We used whole-mtDNA genomes for a detailed phylogenetic reconstruction and to calculate more precise age estimates of the clades and times of expansion. However, whole-mtDNA genomes do not yet provide a comprehensive evaluation of the distribution of each clade, where the much higher number of HVS-I sequences remains essential. Furthermore, the whole-mtDNA genomes do not cover enough of the hypothetical source and sink populations for a founder analysis approach, and we therefore also used HVS-I data for this. 

We constructed a phylogenetic tree using the reduced-median algorithm [57], resolving reticulations by hand on the basis of the relative frequency of the mutations involved [53]. We used the GeneSyn [58] software to first convert all available whole-mtDNA L0 genomes into variants and then convert the variants into a binary file for the Network 4.6 software. Individual genomes are indicated in the reconstructed tree (File S1) and Table S2. Sequences by Gonder et al. [59] were provisionally included, but dropped from most subsequent analyses due to probable artefacts [28]. These data indicated branches of interest in Tanzania amongst a few samples and we placed these on the tree with the diagnostic positions, but not the (potentially erroneous) private mutations included. These branches were excluded from age estimates. Some sequences, mainly those generated from next-generation sequencing, displayed several ambiguous characters and were excluded from the analyses. 

We re-evaluated the timescale for African mtDNA evolution using the mtDNA clock of Soares et al. [53], corrected for purifying selection and tested against the well-established colonization times of America and Oceania [53,60]. This methodology has recently also been applied to the mtDNA tree of other mammalian species [61], and this human mtDNA clock is in very good agreement with the recent recalibration using ancient mtDNA samples [62]. We estimated ages for specific clades in the phylogeny using both the ρ statistic [63] and maximum likelihood (ML), which gave generally similar results. We used ρ (the mean number of mutations from the inferred ancestral haplotype of a given clade) with a mutation-rate estimate for both the whole-mtDNA sequence of one substitution per 3624 years further corrected for purifying selection, and a synonymous mutation rate of one substitution every 7884 years [53], calculating standard errors as before[64]. We estimated branch lengths by ML using PAML 3.13 [65], assuming the HKY85 mutation model with gamma-distributed rates (32 categories), with the same whole-mtDNA genome clock. For the estimation of branches of interest outside L0 (in African L1’6 lineages), we used ML with a tree of 224 random sequences, representing all of the major splits in the human mtDNA tree (samples used are listed in Table S3). Where necessary, we also estimated ages from HVS-I data using the ρ statistic and a rate of 1 in 16,667 years [53]. This was the case for the estimate of the age of L0d3b where the available whole-mtDNA genomes might contain errors [59].

In order to detect signatures of population growth in the L0 haplogroup, we obtained Bayesian skyline plots (BSPs) [66] using BEAST 1.4.6 [67] for the available L0 whole-mtDNA sequences, employing a relaxed molecular clock (lognormal in distribution across branches and uncorrelated between them), a mutation rate of 2.514 × 10^-8^ mutations per site per year for the whole-mtDNA genome [34] and the HKY model of nucleotide substitutions with gamma-distributed rates, assuming a generation time of 25 years. To evaluate how the population growth observed in the L0 haplogroup compared with growth signals overall in African mtDNA, we also used a set of published pan-African whole-mtDNA sequences [28], not focused on any specific haplogroup, and performed four random extractions of 250 sequences. We ran these four alignments of 250 sequences with varying haplogroup composition in BEAST using the parameters described above.

In order to visualize the distribution of L0 and its subclades, we displayed the HVS-I frequency distributions using the Kriging algorithm of Surfer 8. To estimate migration times, we performed a founder analysis [68] of L0 from eastern/central Africa into southern Africa in two ways as described previously [22], one considering all of southern Africa as the sink and the second using only the so-called Bantu “eastern stream” into southern Africa [21,25,69]. We constructed HVS-I networks corresponding to haplogroups L0a, L0b, L0d, L0f and L0k. We searched for sequence matches in our source and sink populations that could represent genetic exchange between areas. However, very recent back migrations are common and they would be displayed as direct matches on the tips of the networks. To avoid this, we used an *f1* criterion that stipulates that the founder type must display a minimal time-depth in the source population represented by at least one derived branch in the clade of the founder sequence [68]. We calculated founder ages for each founder using the accumulated variation in the sink population, a mutation rate of 16,677 years per mutation [53] and the ρ statistic [63]. In order to assess the error in the Bayesian partitioning across the different migration times in a realistic way, we calculated an effective number of samples in each founder [22]. This was obtained by multiplying the number of samples in each founder by a ratio of the variance assuming a star-like network and the variance calculated with the method of Saillard et al. [64]. This value provides the actual number of samples that would be required to obtain the same age estimate and the same precision of the founder considering independence of all mutations in the clade. We scanned the distribution of founder ages by defining equally spaced 200-year intervals [22] for each migration from 0–120 ka. In order to compare the L0 results for southern Africa with the overall southern African pattern, we also performed the founder analyses using all the data (including all clades within L1’6), in the same way as described for L0 alone.

## Results

**Figure 1:**
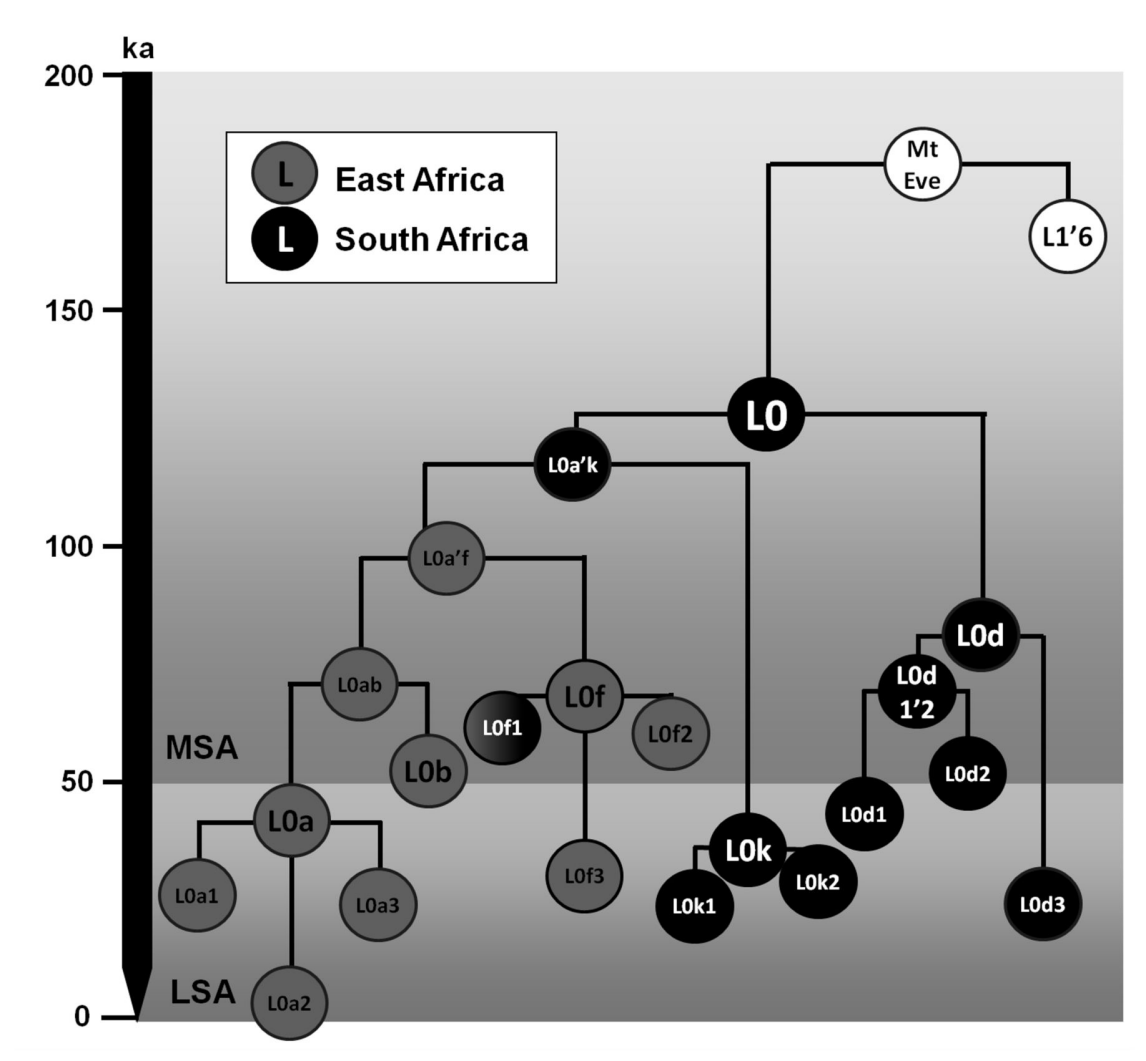
Schematic tree of haplogroup L0 and the root of the human mtDNA diversity. The tree is scaled against the maximum likelihood (ML) age estimates (in ka). Colour scheme for each clade indicates the probable geographic origin (eastern or southern Africa).

 It is extremely challenging to reconstruct population structure going back more than ~60 ka using extant data. Nevertheless, there are very strong phylogeographic patterns in the modern African mtDNA pool that demand interpretation. We present age estimates in Table 1. Although a minority lineage in most populations in terms of frequencies, phylogenetically L0 represents half of the mtDNA variation observed in extant African populations – and indeed worldwide. The modern human mtDNA tree split first at ~180 ka (the age of the “mitochondrial Eve” MRCA) into L0 and a second branch comprising L1-L6 (referred to as L1’6), including the L3 branch that migrated out of Africa 60–70 ka [22] (Figure 1). This L1’6 branch is much more frequent than L0 throughout Africa and has a likely eastern or central African origin, since L5, L6 and L4 are all virtually restricted to eastern Africa while L1 (representing half the clade, phylogenetically) is found in central/west Africa. Its deepest split within L1’6 is between L1 and L2’6 at ~150 ka. L1, dating to ~125 ka, can be inferred to have a central African origin; the central African L1c haplogroup dates to ~80 ka, whereas the more restricted L1b in west Africa dates to only ~30 ka. L2’6, dating to ~130–140 ka, is predominantly eastern African, with more recent splits leading to further predominantly west African clades, the earliest of which is L2 at ~80 ka (Table 1). 

**Table 1:** Age estimates referred to in this study.

**Clade**	**ML whole-mtDNA age estimate (ka)**	**ρ whole-mtDNA age estimate (ka)**	**ρ synonymous age estimate (ka)**
**Human mtDNA root**	178.8 [155.6; 202.2]	185.2 [153.8; 216.9]	174.8 [153.8; 216.9]
**L0**	128.2 [107.9;148.9]	121.3 [99.2;143.7]	131.0 [97.8;164.2]
**L1’2’3’4’5’6**	147.8 [128.2; 167.7]	157.8 [129.8; 186.3]	151.6 [114.3; 189.0]
**L1**	123.8 [106.9; 141.1]	124.8 [100.1; 150.0]	125.1 [90.3; 159.9]
**L1c**	81.9 [70.6; 93.4]	91.7 [73.0; 110.8]	90.9 [65.9; 115.8]
**L1b**	31.0 [22.4; 39.8]	29.3 [15.7; 43.6]	38.2 [12.4; 63.9]
**L2’3’4’5’6**	130.2 [112.6; 147.9]	142.7 [112.0; 174.1]	144.6 [100.8; 188.5]
**L2**	82.8 [70.4; 95.4]	77.0 [56.3; 98.4]	87.9 [51.4; 124.3]
**L5**	109.4 [90.3; 128.9]	108.4 [78.5; 139.3]	142.3 [90.1; 194.6]
**L0d3**	24.4 [14.8; 34.5]	30.4 [16.4; 45.2]	24.6 [8.4;40.9]
**L0a’b’f’k**	119.0 [100.1; 138.2]	121.6 [95.6; 148.3]	122.6 [86.3; 158.9]
**L0k**	35.7 [25.6; 46.1]	36.2 [20.7; 52.5]	42.4 [15.1; 69.7]
**L0a’b’f**	98.7 [82.3;115.4]	86.8 [61.9;112.6]	87.2 [49.7;124.6]
**L0f**	69.3 [56.9; 81.9]	79.6 [63.0; 96.8]	87.2 [61.7; 112.7]
**L0a’b**	70.9 [55.5;86.7]	63.5 [43.2;84.8]	59.2 [29.1;89.2]
**L0b**	51.9 [37.1; 67.4]	62.4 [40.4; 85.5]	66.9 [30.3;103.5]
**L0a**	42.4 [33.0; 52.0]	37.5 [27.5; 47.9]	34.8 [19.5; 50.0]
**L0a1b**	11.9 [7.6; 16.2]	15.8 [7.8; 24.1]	18.7 [2.3; 35.1]
**L0a1a**	13.1 [6.8; 19.6]	12.7 [5.8; 19.9]	17.7 [2.8; 32.7]
**L0a1a2**	6.5 [3.1; 10.1]	8.0 [5.6;10.5]	7.4 [4.4; 10.3]
**L0a2b**	2.7 [0.9; 4.6]	3.6 [1.2; 6.0]	3.6 [0.0; 7.3]
**L0a2a1**	12.0 [6.8; 17.4]	13.4 [7.6; 19.4]	14.0 [2.9; 25.1]
**L0a2a2a**	4.6 [3.0; 6.2]	6.0 [3.7; 8.3]	4.0 [0.7; 7.3]

Estimates were calculated using maximum likelihood and ρ (using both the whole-mtDNA genome and only the synonymous mutations).

**Figure 2:**
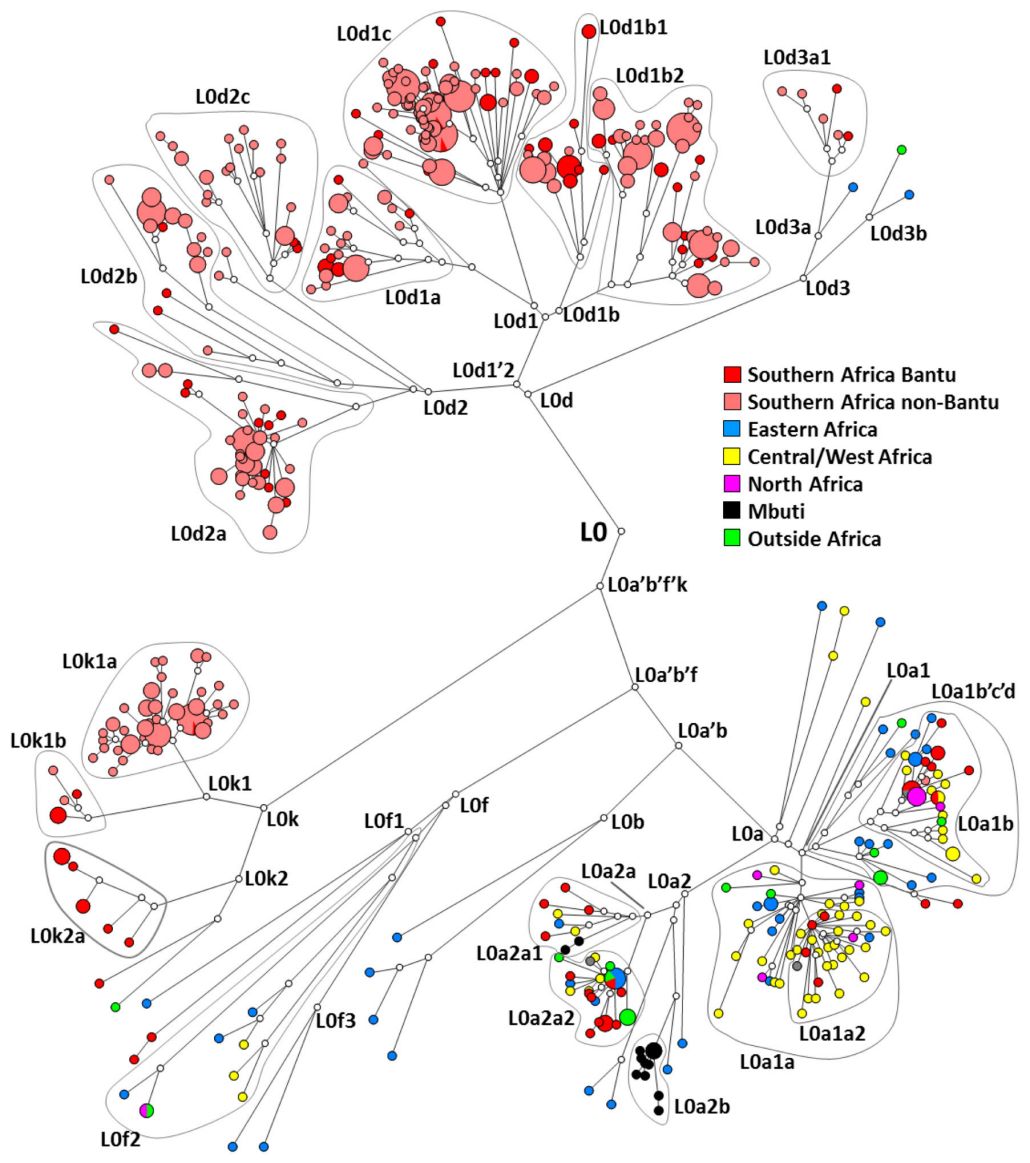
Tree of haplogroup L0 sequences generated using the Network software. The colours indicate the geographic origin of each sample.

**Figure 3:**
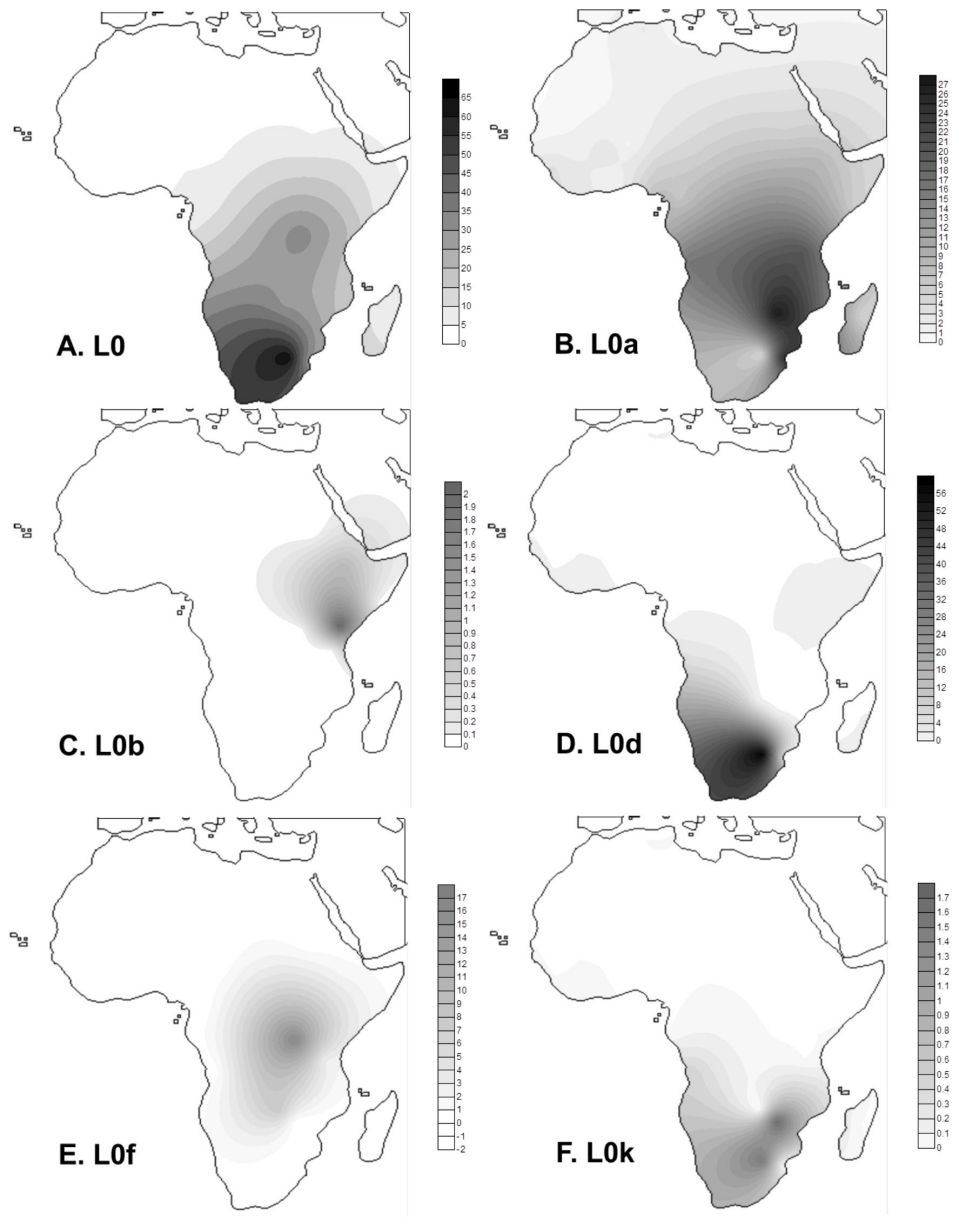
Frequency maps based on HVS-I data for haplogroups L0 (total) (A), L0a (B), L0b (C), L0d (D), L0f (E) and L0k (F).

Figure 2 displays a phylogenetic representation of the whole mtDNA L0 data employed in the analysis. L0, dating to ~130 ka, is far more frequent in the south (Figure 3), in both “Khoesan” and Bantu-speaking groups. The first split is between L0d and the remainder of L0 (L0a’b’f’k), which then splits between L0k and L0a’b’f (Figure 1 and 2). The earliest clades to separate, L0d and L0k, have a largely southern African distribution (Figure 1 and 2), suggesting that L0 as a whole most likely had a southern African origin [29]. However, L0d is divided into two branches, L0d1’2 and L0d3. Whilst the more frequent L0d1’2 is only detected in southern Africa, L0d3 shows evidence of a recent dispersal from southern to eastern Africa: one whole subclade, L0d3b, points to an eastern African specific clade dating to ~7.4 (±4.5 ka, based on HVS-I data) (one sample from Kuwait most probably reflects recent gene flow from eastern Africa) (Figure 2). San, Khoe and Bantu speakers are interleaved within the tree, indicating a broadly common source pool for all three groups (by recent introgression in the case of the Bantu), which one might justifiably refer to as “Khoisan” or “Khoesan” [70,71]. Even so, some early geographic substructure is visible in the gene pool of southern populations [29].

L0a’b’f’k dates to ~120 ka, and splits again to give rise to L0k and L0a’b’f (Figure 1 and 2). L0k does not begin to diversify in the tree until ~40 ka and seems to be rare (Figure 3F), concentrated in the northerly Ju and Khwe. Although L0k1a is almost restricted to Khoesan groups [29], L0k1b is mostly present in Bantu-speaking populations from Zambia [29,72], and this is even more true for L0k2. This study adds two samples from Mozambican Bantu speakers to the two branches of the more common L0k2a. This suggests either gene flow from earlier populations in the area, that no longer survive, into Bantu-speaking populations [29,72] or the disappearance of the clade by drift in extant Khoesan groups after gene flow occurred. Either scenario remains consistent with an ancient origin of L0k in the south.

By contrast, the subclade L0a’b’f, which dates to ~90-95 ka, has a most likely origin in eastern Africa. The frequency distribution of the deepest subclade of L0a’b’f, L0f, is centred on Uganda/Tanzania. L0f, which dates to ~70-80 ka, is quite rare and indeed largely restricted to this region, with minor exceptions. One basal branch, L0f1, shows a deep split between southern Africa Bantu speakers and eastern Africa, L0f3 is represented by two samples from Kenya and Somalia (but also present in Tanzania from the HVS-I data), one presently unclassifiable branch was identified in two Tanzanian samples [59], and L0f2 has a wider distribution that includes central as well as eastern Africa. Higher HVS-I diversity is found in Tanzania (ρ = 3.98 ± 1.11; π = 5.517) than in the rest of eastern Africa (ρ = 3.43 ± 0.95 and π = 5.039), supporting a source there. However, considering the existence of an early South African Bantu lineage in L0f1 and the southern focus of L0f within eastern Africa, an alternative possibility is that L0f as whole has a southern African origin and basal branches disappeared by drift in Khoesan populations (as for L0k) throughout southern Africa.

L0a’b, the sister clade of L0f, dates to ~70 ka and L0b, which is rare and restricted in its distribution, dates to ~50 ka and is centred on Kenya. It therefore seems most likely that L0a’b arose further to the north and east (in the vicinity of Kenya) from a dispersal arriving ~95–70 ka. Since L0 as a whole clearly arose in southern Africa, these patterns most likely track a movement from southern towards eastern Africa sometime between the emergence of L0a’b’f’k ~120 ka and the age of L0a’b, ~70 ka, with a stepping-point in southern eastern Africa ~95-100 ka (L0a’b’f). Pickrell et al. [36] detected an ancient link in genomic data between Khoesan populations and East African populations (Hadza and the Sundawe from Tanzania). It is possible that this is a trace in the genome-wide pattern of this ancient migration, rather than the spread of click-consonant languages, which was probably much more recent. However, no other eastern African data was included in the analysis.

**Figure 4:**
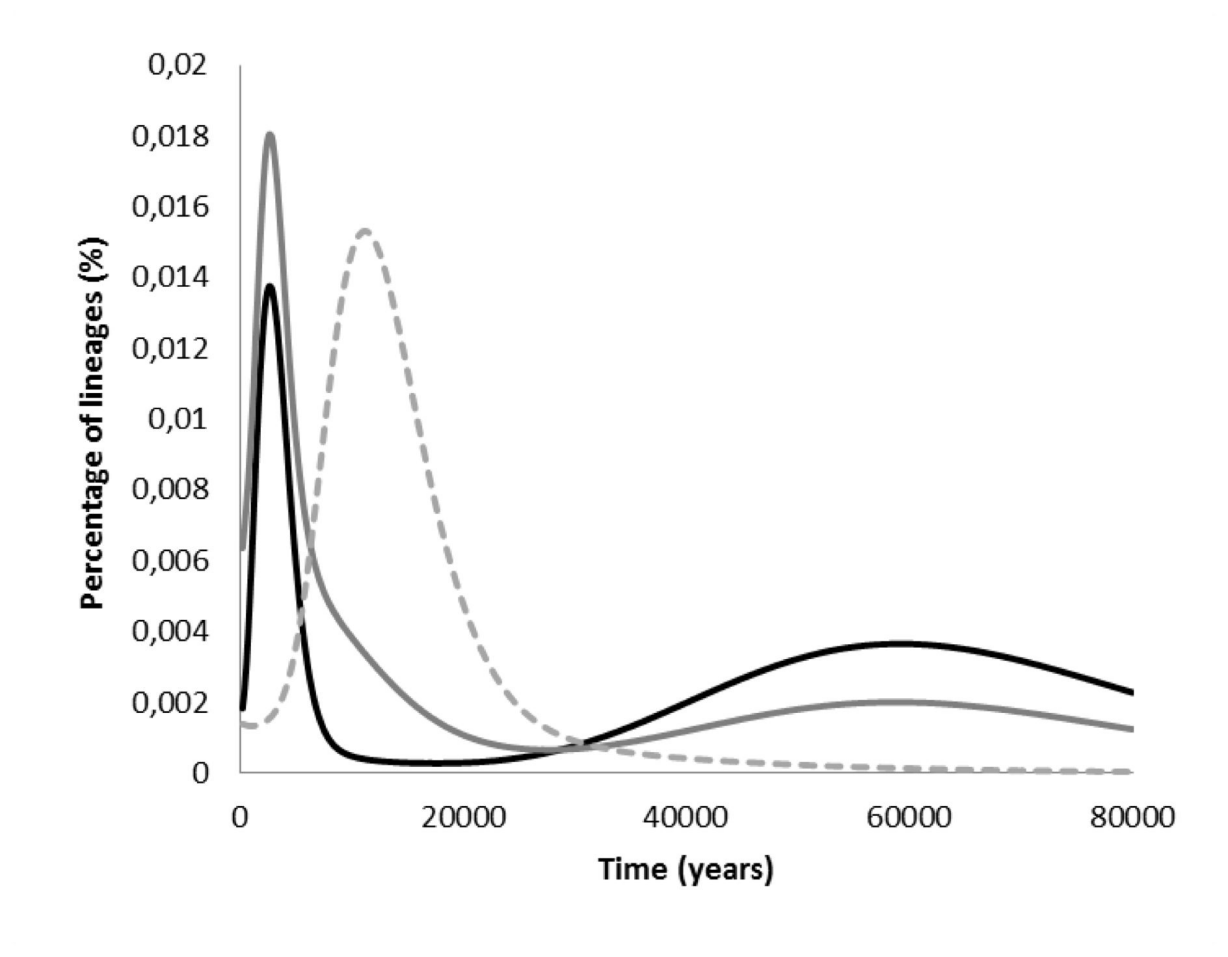
Probabilistic distribution of founder clusters across migration times scanned at 200 years intervals from 0–80 ka, using the *f1* criterion, for southern African L0 sequences (black filled line), for overall southern African mtDNA variation (grey filled line) and for L0 central African sequences considering an eastern African source (dashed line in grey).

The much more common and widespread L0a dates to ~40 ka, and has a more complex distribution, but it most probably has an eastern African origin (considering that the sister clade L0b is completely restricted to eastern Africa and all the L0a sub-clades, L0a1, L0a2 and L0a+95C, display unique basal sub-branches from this region) with subclades extending to west Africa, central Africa, southern Africa, North Africa and the Near East. Although there is a bias towards west African samples in the whole-mtDNA tree, basal branches from eastern Africa are present in all the L0a clades of the present reconstruction (L0a1’4, L0a2, L0a+95C) with only more recent subclades L0a1b, L0a1a, L0a3, L0a2b and L0a2a showing a possible central Africa origin. Comparisons with HVS-I data confirm that an eastern African origin is most likely, as also suggested by the frequency distribution (Figure 3), although the frequency peak in Zimbabwe/Mozambique (~25%) is further to the south than the likely origin due to recent translocations associated with the Bantu dispersals. HVS-I founder ages from eastern Africa to central Africa in L0 indicate a peak of migration of ~11.2 ka, suggesting that the major L0a founders moved into West/central Africa (dashed line in Figure 4) around the Pleistocene/Holocene transition period, when climate conditions improved, and when major expansions are also discernible on the mtDNA record of northern locations namely Southwest Asia [73] and Europe [74]. L3f in eastern/central Africa testifies to the same pattern as L0a [22]. Three HVS-I founders (84% of the L0a lineages in central/west Africa) are responsible for the signal (L0a1*, L0a1+16293 and L0a2*). These results are supported by the whole-mtDNA analysis. L0a1+16293 includes two eastern African clades (L0a1c and L0a1d) and a third (L0a1b) that is mostly central/west African, dating to 12–16 ka. The HVS-I L0a1 root founder corresponds (at least primarily) to L0a1a in the whole-genome tree. This subclade is most frequent in central Africa (although also present in eastern Africa) and dates to ~15 ka, whilst the derived subclade, L0a1a2, is restricted to central/west Africa and dates to ~9–10 ka. Several other lineages within L0a2 might have also moved in this period. L0a2b, dating to ~5-6 ka, is restricted to central African Forest Mbuti but shares a link with eastern Africa dating to more than 15 ka. L0a2a1, dating to ~14 ka, is also mostly central/west African. L0a2a2a, dating to 4–5 ka, is mostly associated with Bantu-speaking populations and a central African origin is also likely for this subclade.

**Figure 5:**
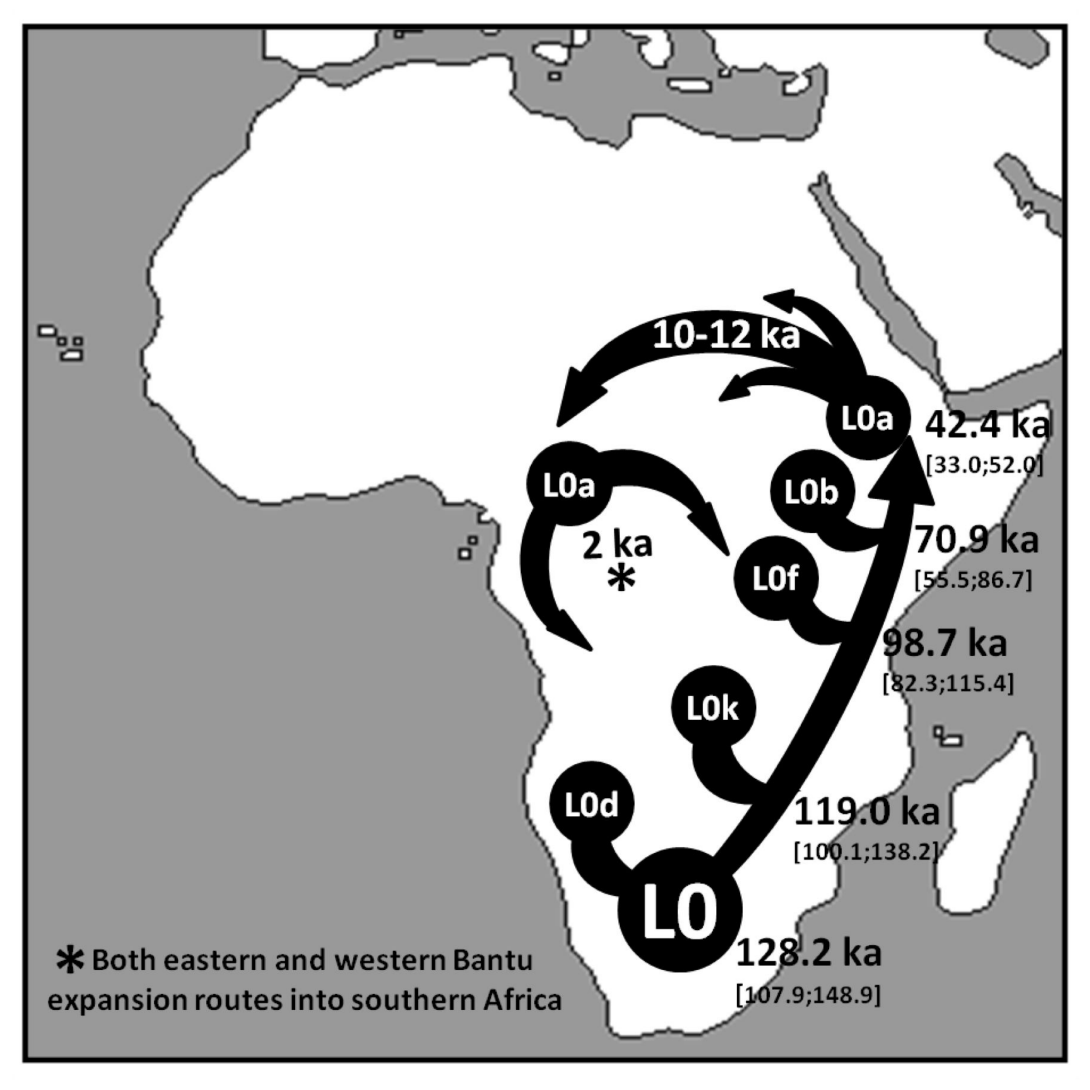
Schematic representation of the major inferred migrations involving mtDNA haplogroup L0.

A founder analysis for the whole of L0, using HVS-I, data shows a clear peak at ~2 ka for both the overall southern Africa scan and also when considering only the eastern Bantu route (Figure 4 – for clarity only the overall South African scan is shown). This recent peak is generated by the founder ages of the L0a subclades in southern Africa linked to the Bantu expansion, most likely from an origin in the Great Lakes region of eastern Africa. L0a1+16293 (most probably L0a1b in the whole-sequence tree) and L0a2 (most probably L0a2a1 and L0a2a2a) are the founder lineages that are primarily responsible for this signal. These lineages moved from eastern Africa into central Africa in the late Pleistocene/early Holocene and were then integrated with the populations that would later become the dispersing Bantu. This points to the extraordinary complexity of even the baldest summary of mtDNA phylogeography in Africa: the human root of haplogroup L perhaps originated in central Africa ~180 ka; L0 originated in southern Africa ~130 ka; L0a’b’f arose in eastern Africa ~95 ka; and L0a evolved in eastern Africa ~40 ka, from where some lineages spread into central Africa during Late Pleistocene/early Holocene and the further into southern Africa ~2 ka with the Bantu expansion (Figure 5). 

**Figure 6:**
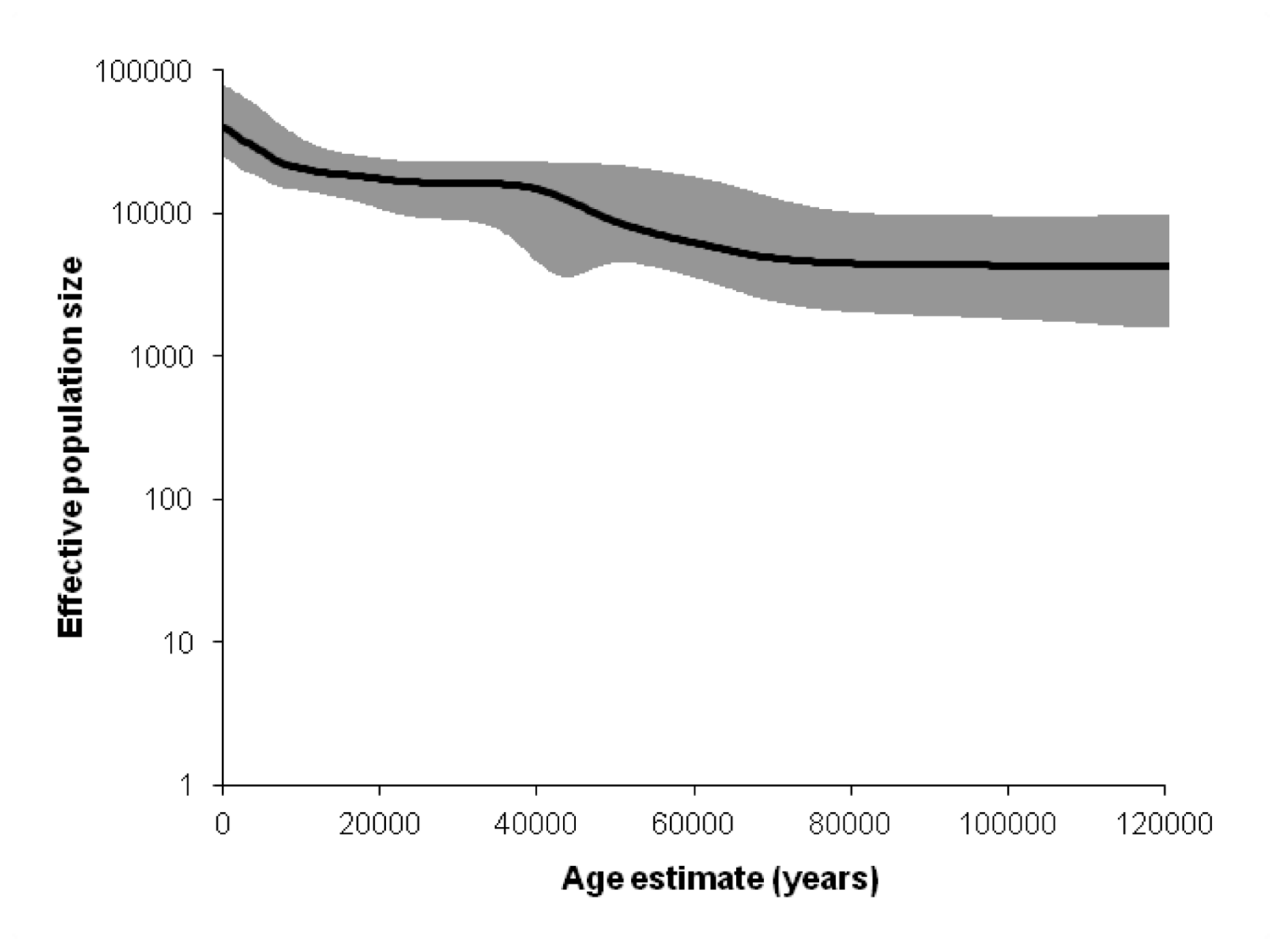
Bayesian skyline plot (BSP), indicating hypothetical effective population size through time, based on data from the entire L0 haplogroup.

This very recent dispersal from eastern to southern Africa is reflected in the BSP analysis of population size, where the main increase for L0 is observed between 0.5 and 5.7 ka, leading to an increment of just above 2 times (Figure 6, Table 2), similar to the pattern obtained for a previous L0 BSP analysis [75]. Unlike the pattern observed in L3, and even with U6 in North Africa [22,34], we do not see strong Late Glacial or postglacial signals in L0, even though L0a was probably involved in some dispersals in this period. We do see a small increment just before ~40 ka in L0 (Figure 6, table 2), which we also observed in L3 [22] (Table 2). Geographically res**t**ricted BSP analyses of eastern and southern Africa also display the increment only in the last 4–5 ka (Figures S2), although some hint of an increment after 20 ka could be discerned in eastern Africa, suggesting that the lack of a postglacial signal may be due to low resolution. 

**Table 2:** Peaks of rate of population size change through time.

**Data**	**Peak**	**Range of Increment**	**Increment ratio**
**L0**	46.6	45.5-47.2	1.23
	0.3	0.0–5.7	2.01
**L3**	40.3	38.5–43.3	2.89
	10.9	9.1–12.4	4.59
	3.3	1.7–4.3	9.52
**Random1**	46.1	45.8–47.2	1.11
	12.3	10–13.8	2.74
	3.8	2.3–6.5	1.99
**Random2**	11.2	8.2–12.6	3.54
	3.5	2.6–6.2	1.58
**Random3**	39.4	39.1–42.0	1.39
	12.3	10.9–13.8	3.07
	3.8	2.1–5.3	2.73
**Random4**	46.5	45.1–47.7	1.13
	11.8	9.5–13.0	3.01
	4.3	2.6-5.8	3.41

The values were obtained from the BSPs for the L0 data and four random samplings across the mtDNA tree of Behar et al. [28] for periods of time where the rate of population size increase was of at least one individual per 100 individuals in a period of 100 years. Results from L3 [22] are included for comparison.

An increase between 13.5 and 16.3 was, however, observed in the BSP for southern Africa, mainly represented by L0d samples, suggesting that southern Africa also underwent demographic changes at this period. The BSP of L0 for central Africa (Figure S2) indicates a single increment, beginning at ~9 ka and lasting till the present (8.6–0.0 ka) with a population increment higher than 10 times, possibly a hybrid signal between postglacial expansions and the Bantu expansion. This is effectively the same signal detected when analysing L0a as a whole, since L0a was the L0 subclade involved in these events (Figure S2). An analysis of southern African haplogroups L0d and L0k also indicates a pattern similar to the one in the overall southern data. However, somewhat surprisingly, the postglacial signal is not visible when analysing L0d/L0k in the Khoesan alone but is visible in the L0d/L0k sequences present in the Bantu speakers (Figure S2). This illustrates both the fact that a substantial signal has been lost in Khoisan populations, but also that some of that signal persists in Bantu-speaking populations that interacted with them, with L0k2 being one example [29,72].

We finally compared the L0-specific BSP results with an estimated overall pattern for sub-Saharan Africa. The results with various random subsets of African whole-mtDNA genomes (Figure S2), always showed a large increment in the last few millennia (Table 2), an increment at the Pleistocene/Holocene transition (observed in both L3 and U6), and usually an increment starting before ~40 ka (Figure 5). This latter age is in the vicinity of the very imprecise and contested Middle to Late Stone Age transition. It is worth mentioning that an increase in the effective population size might not necessarily signal population growth. An increase in population structure could mimic a signal of population growth in the BSP [76].

## Discussion

Genetic and archaeological discussion has focused for many years on the possibility that the appearance of AMH ~200 ka was accompanied by a speciation bottleneck, partly inspired by the similarities of the age of “mitochondrial Eve” to the age of the earliest AMH fossils. Although the view has frequently been expressed that such a bottleneck existed in MIS 6, ~190–130 ka, and it has sometimes been assumed (and leant implicit support) in demographic modelling [77], clear genetic evidence has been lacking, and although archaeologically plausible [6,78] recent autosomal studies render anything more than a very shallow bottleneck unlikely [31]. 

We would expect a deeper coalescence time for the autosomes and X-chromosome (~1.5 Ma and 1.0 Ma, respectively) in comparison with the haploid mtDNA and Y-chromosome lineages, since the effective population sizes are four and three times greater, respectively. However, since for mtDNA the coalescence time is below 200 ka, the scale of the difference is in fact greater than we would anticipate. This can, however, be explained by invoking a structured archaic population in Africa [79]. Such a scenario is clearly plausible on the basis of the fossil record, since human remains are found throughout northern, eastern, central and southern Africa between the time of divergence from *Homo heidelbergensis*, ~600 ka, and the emergence of fully modern *Homo sapiens* ~200 ka, with no clear discontinuity or speciation event [2,6]. Whilst there is evidence in the archaeological record for an expansion of *Homo sapiens* alongside Mode 3 technology (which originated much earlier, 240–280 ka) with the onset of MIS 5, ~130 ka, there is no evidence pointing to a bottleneck as such. In the absence of a tight speciation bottleneck, the similarity in the timing of the TMRCA of mtDNA and the first appearance of AMH fossils can be no more than a coincidence, although it is an indicator of the low human population size during MIS 6. Given this, the several lines of evidence highlighting the high levels of autosomal diversity in southern Africa may be pointing back to a time earlier than the emergence of AMH, and indeed the search for a single origin for AMH may prove to be a mirage [1,2]. Modern *Homo sapiens* may indeed have arisen by “multiregional evolution” within Africa [80], even if perhaps driven selectively by the expansion of parts of the brain, such as the temporal lobes [30,81].

The mtDNA tree is geographically structured at the deepest, basal level, with L0 branching in southern Africa and L1’6 in eastern/central Africa. This implies a dispersal between one region and the other, and clearly “mitochondrial Eve”, carrying the MRCA of human maternal lineages, must have lived in a specific location ~180 ka, but as noted before [21,28] that location is difficult to recover phylogeographically in the absence of the discovery of any further deep lineages. A “centre-of-gravity” argument might suggest an origin in central Africa, but in any event, if several populations coalesced at various times during MIS 6, then it is not even clear that the MRCA of mtDNA would trace to a modern, rather than archaic, human population. Even so, the distribution of nucleotide diversity in mtDNA HVS-I throughout Africa does suggest that central Africa is the most diverse region [82]. Furthermore, a central African source for modern Y-chromosome variation is supported by evidence that the deepest-rooting lineages have been found in west-central Africa [16,17], which might even provide some measure of agreement with the most up-to-date autosomal evidence [12]. A further hint is provided by the distribution of “human-specialist” parasites, which are mainly concentrated in central/West Africa, which has been interpreted as suggesting that early human evolution took place there [83]. Given these different lines of evidence, a Central/West African source seems increasingly plausible.

What does seem clear is that L0 most likely arose in southern Africa amongst the distant ancestors of the modern-day Khoe and San populations, contrarily to the sister clade L1’6 that had a northern origin. This result is indirectly supported by genome-wide data that indicate an early split between Khoesan populations and northern populations more than 100 ka [13–15]. It is intriguing that the deepest geographically restricted branches of the tree – L0, L1 and L2’6 – evidently originated in southern, central and eastern Africa respectively, and all date to ~130 ka, close to the onset of MIS 5 and the beginning of the megadroughts in central Africa, and also the time at which *Homo sapiens* becomes much more visible in the archaeological record, with the widespread appearance of Mode 3 industries throughout Africa [6,84]. By this time, too, the archaic specimens in the fossil record finally disappear [78].

This lends weight to the suggestion that the increased aridity in central Africa might have actually created the potential for demographic expansion into this zone by thinning the rainforest [52] – Middle Stone Age sites are frequently associated with open woodland in MIS 6, and human populations became increasingly mobile with the onset of MIS 5 [78]. We can envision dispersals of the bearers of the L0 and L1’6 dispersing from a “centre of gravity” in central Africa northwards through the tropical zone, and ultimately into eastern Africa (the oldest specific eastern African specific mtDNA lineage, L5, dates to the start of the eastern African moist phase ~110 ka [52]). They perhaps dispersed simultaneously southwards, towards the southern coastal refugial regions [85], most likely settling the inland regions when the climate improved in southern Africa ~90 ka (roughly matching the emergence of the southern African haplogroup L0d), after which modern human remains are found e.g. at Border Cave [6]. 

This would post-date the appearance in southern Africa of what have been described as “key elements” of modern human behaviour (possible bladelets, shell-fishing and potentially even the symbolic use of red ochre) at Pinnacle Point on the South African coast, ~165 ka [45,47], but whether these were produced by “anatomically modern” people is unclear at present. The oldest direct evidence of AMH remains in southern Africa are from Klasies River Mouth, in deposits that date back to ~120 ka [69]. Whatever the precise details, this earliest split amongst human mtDNAs signals the earliest dispersal detected using genetic data amongst early-modern humans. 

The combined whole-genome and HVS-I mtDNA database suggests that none of the remaining mtDNA diversity (in haplogroup L1’6) supports such an early presence for L0’s sister clade in the south of the continent. In fact, there is no clear evidence of any lineages within L1’6 dating to earlier than the late Holocene in southern Africa (Figure 4) (*cf*. Behar et al [28]). Genome-wide analysis also indicates that admixture between Khoesan and non-Khoesan only began ~1.2 ka [36]. The presence of L1c, L2b, L3d and L3e from west-central Africa into the ancestors of some Khoe-speaking herding groups [26,86,87] most probably simply indicates high levels of intermarriage with Bantu immigrants. Although dispersal of pastoralism from eastern Africa prior to the Bantu dispersals has been suggested on archaeological [70] and Y-chromosome [88] grounds, the eastern African L0a signature is so far absent from these groups and prominent rather in eastern Bantu speakers in Mozambique and South Africa [21,25,86]. L0 is therefore the sole link between eastern Africa (and the northern part of the continent more generally) and southern Africa in the Pleistocene.

L0a’b’f provides the first unambiguous signal of a long-range modern human dispersal, from southern to eastern Africa, ~120–75 ka. Moreover, as suggested by the distribution of L0f, L0a’b’f looks likely to have been present in the southern part of eastern Africa by ~95 ka, with L0a’b appearing in the vicinity of the Horn of Africa by ~75 ka. This timing also corresponds roughly to renewed megadrought conditions in central Africa, beginning ~115 ka, which may, as mentioned above, have facilitated the expansion of human groups by creating a more open landscape in the tropical rainforest zone [52]. Again, this may have sucked some human groups (carrying L0a’b’f) in from southern Africa, whilst others (carrying L0d and L0k) became confined to southern coastal refugia [85]. The end of the arid period and the renewed spread of rainforest in tropical Africa ~75 ka may have provided the impetus for the final push into eastern Africa and the advent of L0a’b. Another possible scenario is that L0f might actually have arisen in southern Africa and the expansion of L0a’b in eastern Africa might represent a direct, second, more recent migration from southern Africa, after ~70 ka.

Intriguingly, the time of arrival of L0a’b in eastern Africa is close to the time of expansion of haplogroup L3. L3 arose in eastern Africa and was the first mtDNA lineage to undergo a major population expansion, both westwards into central Africa and eastwards out of Africa, ~60 ka [24,33]. One possibility is that these dispersals may have been connected to the arrival the people carrying L0a’b. Perhaps, given that the earliest evidence for *symbolically mediated* behaviour (pointed to in the archaeological record by engraving or ornamentation, as opposed to other, less clear-cut “markers of modernity” such as microlithic industries and shell-fishing) may have been present in southern Africa, in the form of incised ochre at Pinnacle Point, ~165 ka [45], it is possible that this aspect of modern behaviour was incubated in southern Africa, and spread north and eastwards alongside the L0a’b’f lineage, passing its legacy to eastern populations carrying L3. The same dispersal might conceivably have carried the use of *Nassarius* bead ornamentation to North Africa, where they are found amongst the Aterian industries by ~85 ka [42,43] (whose descendants probably did not survive to the present day [20,34]), although evidence for naturally perforated *Glycymeris* beads (as well as ceremonial burial sites involving use of ochre as a pigment) in the AMH Levantine sites of Skhul and Qafzeh dates to >90 ka [89]. 

This, by the way, appears to have been significantly earlier than the putative dispersal of the strikingly modern Howiessons Poort (HP) industry from southern to eastern Africa. HP appears to have arisen indigenously in South Africa ~65 ka from the Still Bay industry, which dates to 70–74 ka [90] and to have spread rapidly to eastern Africa by ~60 ka [91]. It seems unlikely that this can be accounted for by the pattern we see in L0, which seems to disperse northwards too early, but given the imprecision of genetic dating, it is not a possibility that should be completely ruled out at this stage. In fact, an origin for L0a’b’f in the south (as discussed above) and a later entrance of L0a’b, dating to shortly after ~70 ka, into eastern Africa, could provide a potential signal of a dispersal from south to east at the appropriate time. As an alternative, perhaps the HP innovations spread northwards by way of channels opened up through central Africa by the earlier dispersals associated with L0a’b’f. 

A much more recent dispersal from south to east is also indicated within L0d. L0d3 splits into two subclades, L0d3a and L0d3b. L0d3a is found mainly in southern Africa, with two occasional examples in Kenya and Somalia. Again, considering that the more common sister-clade L0d1’2 is entirely South African and L0d3b is mostly Southern African, it is much more parsimonious that L0d3 is South African. However L0d3b is restricted to eastern Africa, and is found primarily in the Tanzanian Sandawe, who speak a click-consonant language [59,92]. This clade is actually the only specific clade that exists in the Sandawe and considering the Southern origin of L0d in Khoesan groups establishing a connection with click-languages is inevitable. The age if this subclade is 7.4 ± 4.5 ka, based on HVS-I data (complete genomes are dubious). This suggests that the dispersal into eastern Africa was most likely between the age of L0d3 in South Africa (~25 ka) and the age of L0d3b dating to 7.4 ka. The data suggests that click-languages were carried to East Africa in the last 20 ka and possibly as recent as the Holocene period rather than being part of a very ancient distribution that included East Africa [71,92], before contracting during the Holocene. More probable is that they were restricted to southern Africa and expanded to only a very limited extent during the last 20 ka. This might be perhaps more consistent with possible similarities between the Sandawe and Khoe languages [71], which would be unlikely to have persisted since the late Pleistocene, and with autosomal patterns which indicate only a small “southern” influence in the Sandawe and Hadza [11,12].

This study highlights both the complexity and the potential of the phylogeographic study of African mtDNA lineages. There has been much discussion about whether climate or culture was the main factor shaping early modern human dispersals, and we have suggested previously that the expansion of haplogroup L3 from eastern Africa, which led to dispersals both across Africa and out of Africa in the period 70–50 ka, may have been driven principally by the proposed improvement in climate in eastern Africa at that time [22,50]. Our analysis here suggests that both may have been crucial. We have shown that there are highly suggestive correlations between major episodes of climate change in Africa and early modern human dispersals. Both the population expansions and the associated wider dispersal of mtDNA lineages ~60 ka attest to a large and more interconnected human population at around this time that may have been more readily able to retain cultural innovations for future generations [49], thus lending some *post-hoc* support to a kind of “human revolution” model based on the establishment of broad-scale social networks, despite the previously piecemeal appearance of modern human features [1]. 

## Supporting Information

Figure S1 **Geographic distribution of the new whole mtDNA genomes produced in this study.**
(PDF)

Figure S2 **Bayesian Skyline Plot (BSP) for mtDNA haplogroup L0 and African random datasets.**
(PDF)

File S1 **Reconstructed phylogeny of mtDNA haplogroup L0.**
(XLS)

Table S1 **HVS-I datasets used in this study.**
(PDF)

Table S2 **Description of the complete mitochondrial sequences from haplogroup L0 used in this study.**
(PDF)

Table S3 **224 random sequences used in an overall African mtDNA tree.**
(PDF)
