# Supplementary material for: The First Modern Human Dispersals across Africa
Source: PLoS One. 2013 Nov 13;8(11):e80031. doi: 10.1371/journal.pone.0080031 (PMC3827445; doi:10.1371/journal.pone.0080031)

Figure S1. Geographic distribution of the new whole mtDNA genomes produced in this study. Colors relate to the geographic sub-divisions used in the analysis (yellow for Central/West Africa, blue for East Africa and red for South Africa).

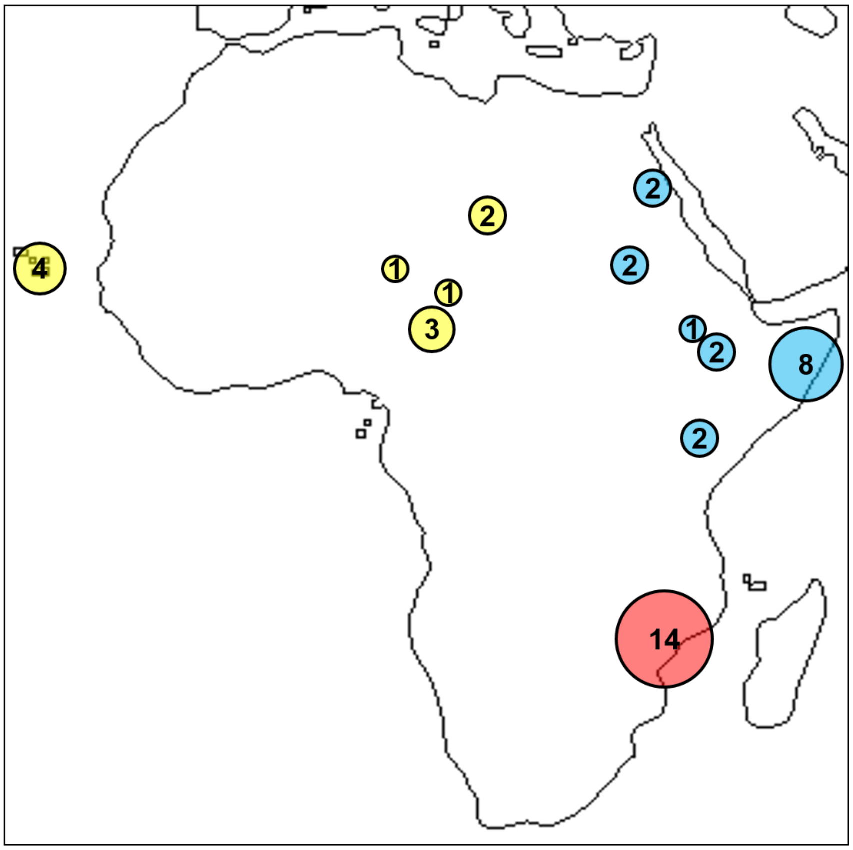

Supplement: Figure S1 — Geographic distribution of the new whole mtDNA genomes produced in this study. (PDF) [file pone.0080031.s001.pdf]
