# Supplementary material for: The First Modern Human Dispersals across Africa
Source: PLoS One. 2013 Nov 13;8(11):e80031. doi: 10.1371/journal.pone.0080031 (PMC3827445; doi:10.1371/journal.pone.0080031)
Supplement: Figure S2 — Bayesian Skyline Plot (BSP) for mtDNA haplogroup L0 and African random datasets. (PDF) [file pone.0080031.s002.pdf]

Figure S2. Bayesian Skyline Plot (BSP) for mtDNA haplogroup L0 in eastern Africa (A), southern Africa (B) and central/western Africa (C), for mtDNA haplogroup L0a (D), mtDNA haplogroups L0d and L0k (E), mtDNA haplogroups L0d and L0k in South Khoesan-speaking populations (F), mtDNA haplogroups L0d and L0k in South Bantu-speaking populations (G), and in four random African mtDNA datasets (H-K).

(A)

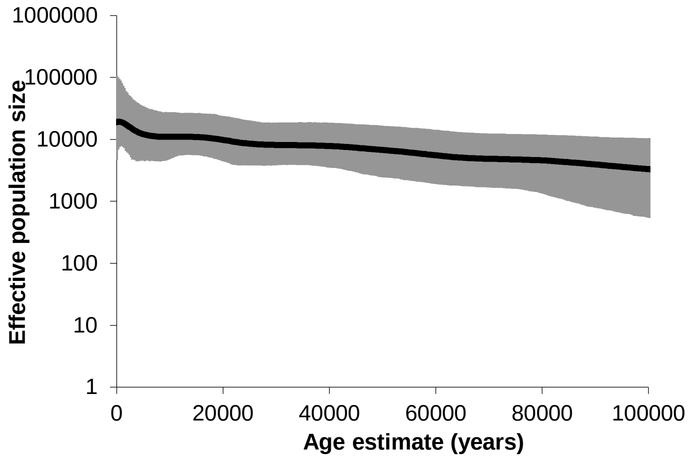

(B)

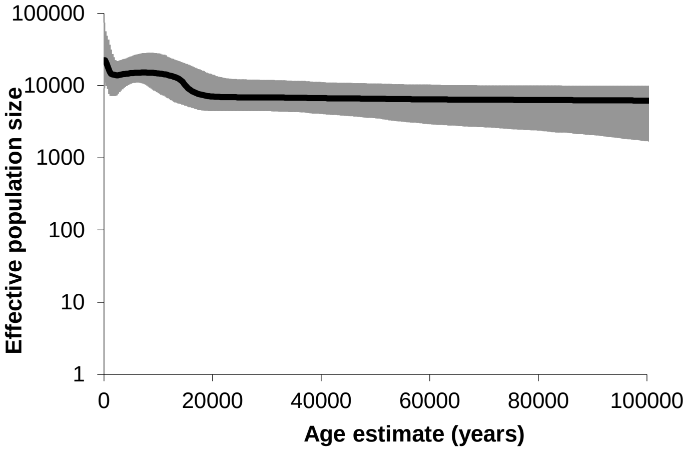

(C)

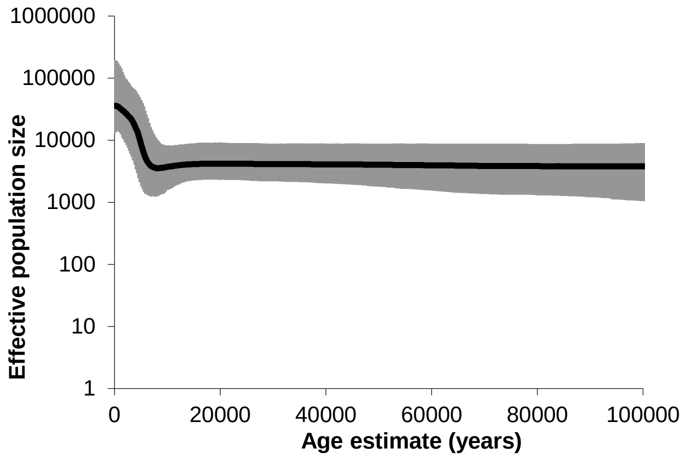
1000000
100000
10000
1000
100
10
1
Effective population size
0
20000
40000
60000
80000
100000
Age estimate (years)

(D)

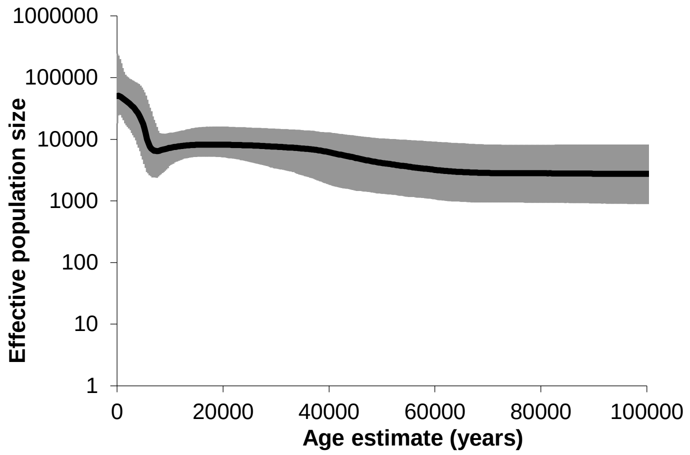
1000000
100000
10000
1000
100
10
1
Effective population size
0
20000
40000
60000
80000
100000
Age estimate (years)

(E)

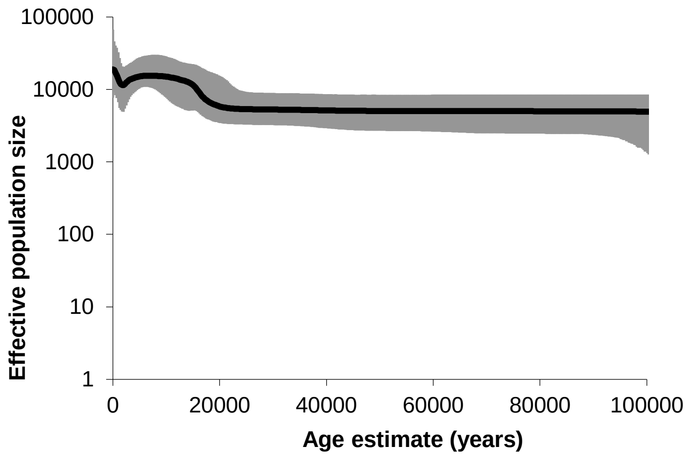
100000
10000
1000
100
10
1
Effective population size
0
20000
40000
60000
80000
100000
Age estimate (years)

(F)

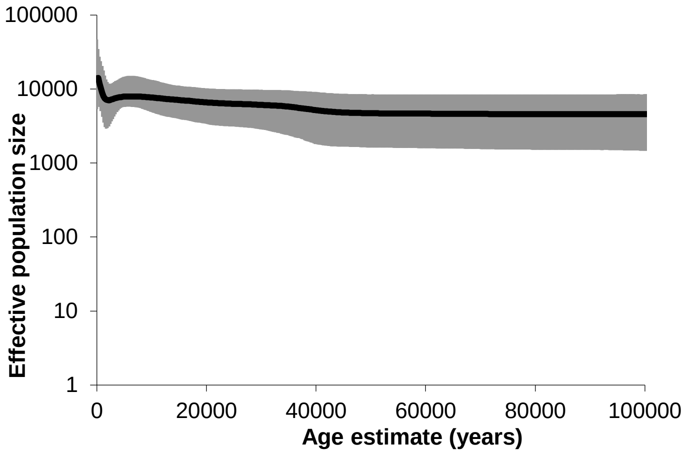
Effective population size
100000
10000
1000
100
10
1
0
20000
40000
60000
80000
100000
Age estimate (years)

(G)

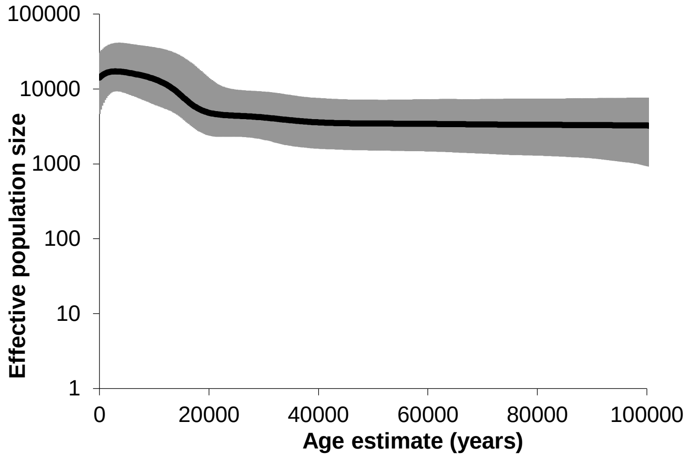
Effective population size
100000
10000
1000
100
10
1
0
20000
40000
60000
80000
100000
Age estimate (years)

(H)

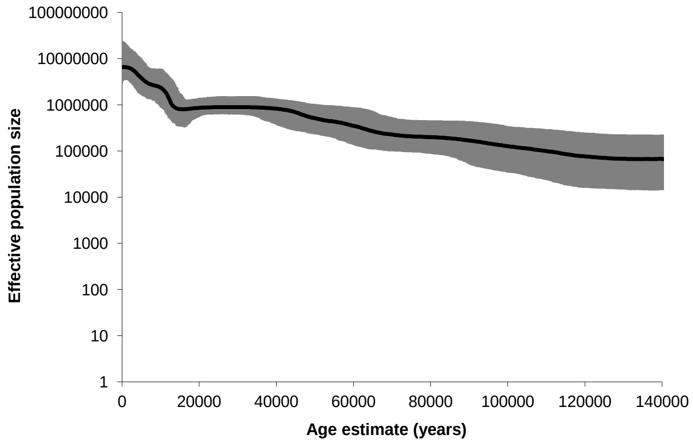
Effective population size
100000000
10000000
1000000
100000
10000
1000
100
10
1
0
20000
40000
60000
80000
100000
120000
140000
Age estimate (years)

(I)

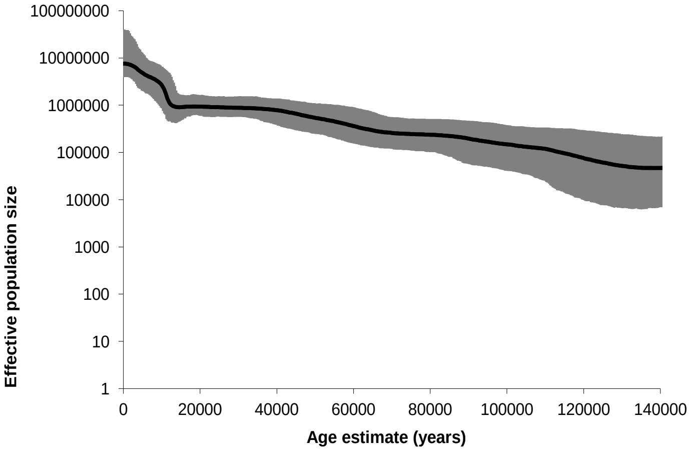
100000000
10000000
1000000
100000
10000
1000
100
10
1
Effective population size
0
20000
40000
60000
80000
100000
120000
140000
Age estimate (years)

(J)

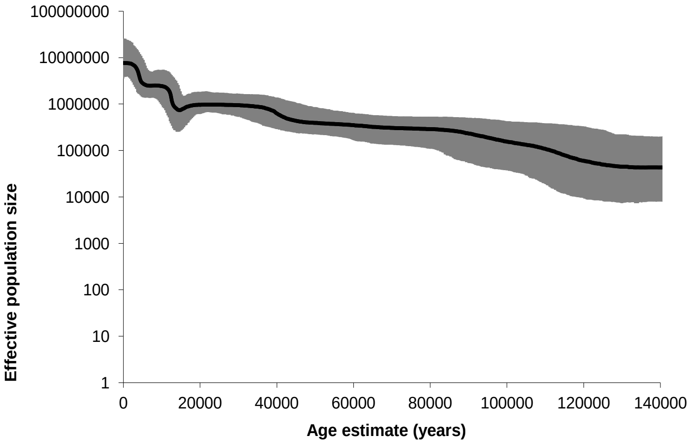
100000000
10000000
1000000
100000
10000
1000
100
10
1
Effective population size
0
20000
40000
60000
80000
100000
120000
140000
Age estimate (years)

(K)

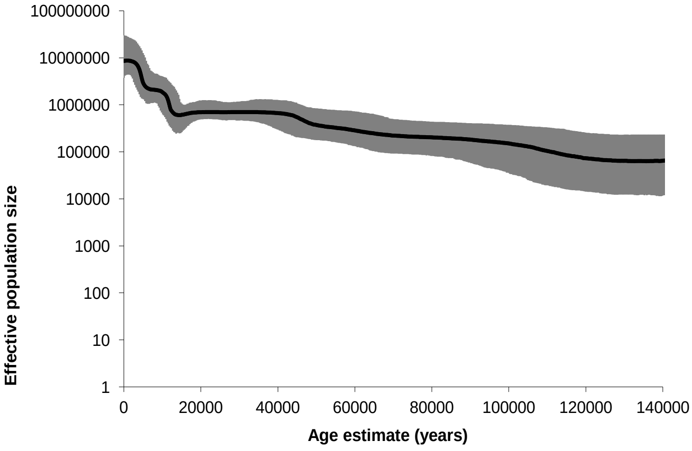
100000000
10000000
1000000
100000
10000
1000
100
10
1
Effective population size
0
20000
40000
60000
80000
100000
120000
140000
Age estimate (years)
