# Supplementary material for: The First Modern Human Dispersals across Africa
Source: PLoS One. 2013 Nov 13;8(11):e80031. doi: 10.1371/journal.pone.0080031 (PMC3827445; doi:10.1371/journal.pone.0080031)
Supplement: Table S1 — HVS-I datasets used in this study. (PDF) [file pone.0080031.s004.pdf]

Table S1. HVS-I datasets used in this study.

General location	Location	Number	Reference
**North Africa**	Algeria	47	[1]
	Egypt	516	[2-5]
	Egypt (Berbers)	78	[6]
	Lybia	269	[7]
	Lybia (Tuareg)	129	[8]
	Morocco	438	[9-14]
	Morocco (Berbers)	278	[1,6,13]
	Morocco (Arabs)	443	[1,9]
	Tunisia	164	[1,14,15]
	Tuareg	22	[16]
**East Africa**	Ethiopia	518	[17-19]
	Ethiopia (Jews)	41	[20]
	Kenya	349	[16,21,22]
	Somalia	185	[16,19,22]
	Sudan	178	[2,19]
	Tanzania	168	[23,24]
	Tanzania (Click consoant-speakers)	210	[23,24]
**Central Africa**	Cameroon	609	[25-27]
	Cameroon (Pygmy)	241	[24,26]
	Congo (Pygmy)	60	[16,26]
	Central Africa Republic (Pygmy)	56	[26]
	Equatorial Guinea	45	[28]
	Gabon	831	[26]
	Gabon (Pygmy)	115	[26]

	Nigeria	1425	[16,25,27]
	Rwanda	42	[29]
**West Africa**	Burkina Faso (nomad pastoralists)	81	[25]
	Burkina Faso (Tuareg)	38	[30]
	Sierra Leone	276	[31]
	Ghana	238	[27]
	São Tomé and Princípe	153	[28,32]
	Cabo verde	291	[33]
	Senegal	280	[13,16,34]
**Sahel**	Chad	118	[16,25]
	Mali	261	[25,35,36]
	Mali (Tuareg)	22	[30]
	Niger	38	[16,25]
	Niger (Tuareg)	31	[30]
	Niger (nomad pastoralists)	127	[25]
	Mauritanea	94	[13,35]
	West Sahara	84	[1,13]
**South Africa**	Angola	519	[1,37,38]
	Kalahari Desert (Khoesan)	19	[16]
	Mozambique	416	[39,40]
	South Africa	563	[41]
	South Africa (Khoesan)	92	[24,42]
	Zambia	78	[43]
	Zimbabwe	59	[29]

References

1. Plaza S, Calafell F, Helal A, Bouzerna N, Lefranc G, et al. (2003) Joining the pillars of hercules: mtDNA sequences show multidirectional gene flow in the western Mediterranean. Annals of Human Genetics 67: 312-328.
2. Krings M, Salem AH, Bauer K, Geisert H, Malek AK, et al. (1999) mtDNA analysis of Nile River Valley populations: A genetic corridor or a barrier to migration. American Journal of Human Genetics 64: 1166-1176.
3. Saunier JL, Irwin JA, Strouss KM, Ragab H, Sturk KA, et al. (2009) Mitochondrial control region sequences from an Egyptian population sample. Forensic Science International-Genetics 3: E97-E103.
4. Stevanovitch A, Gilles A, Bouzaid E, Kefi R, Paris F, et al. (2004) Mitochondrial DNA sequence diversity in a sedentary population from Egypt. Annals of Human Genetics 68: 23-39.
5. Kujanova M, Pereira L, Fernandes V, Pereira JB, Cerny V (2009) Near eastern neolithic genetic input in a small oasis of the Egyptian Western Desert. American Journal of Physical Anthropology 140: 336-346.
6. Coudray C, Olivieri A, Achilli A, Pala M, Melhaoui M, et al. (2009) The Complex and Diversified Mitochondrial Gene Pool of Berber Populations. Annals of Human Genetics 73: 196-214.
7. Fadhlaoui-Zid K, Rodriguez-Botigue L, Naoui N, Benammar-Elgaaied A, Calafell F, et al. (2011) Mitochondrial DNA Structure in North Africa Reveals a Genetic Discontinuity in the Nile Valley. American Journal of Physical Anthropology 145: 107-117.
8. Ottoni C, Martinez-Labarga C, Loogvali EL, Pennarun E, Achilli A, et al. (2009) First Genetic Insight into Libyan Tuaregs: A Maternal Perspective. Annals of Human Genetics 73: 438-448.
9. Rhouda T, Martinez-Redondo D, Gomez-Duran A, Elmtili N, Idaomar M, et al. (2009) Moroccan mitochondrial genetic background suggests prehistoric human migrations across the Gibraltar Strait. Mitochondrion 9: 402-407.
10. Brakez Z, Bosch E, Izaabel H, Akhayat O, Comas D, et al. (2001) Human mitochondrial DNA sequence variation in the Moroccan population of the Souss area. Annals of Human Biology 28: 295-307.
11. Falchi A, Giovannoni L, Calo CM, Piras IS, Moral P, et al. (2006) Genetic history of some western Mediterranean human isolates through mtDNA HVR1 polymorphisms. Journal of Human Genetics 51: 9-14.
12. Harich N, Costa MD, Fernandes V, Kandil M, Pereira JB, et al. (2010) The trans-Saharan slave trade - clues from interpolation analyses and high-resolution characterization of mitochondrial DNA lineages. Bmc Evolutionary Biology 10.
13. Rando JC, Pinto F, Gonzalez AM, Hernandez M, Larruga JM, et al. (1998) Mitochondrial DNA analysis of Northwest African populations reveals genetic exchanges with European, Near-Eastern, and sub-Saharan populations. Annals of Human Genetics 62: 531-550.
14. Turchi C, Buscemi L, Giacchino E, Onofri V, Fendt L, et al. (2009) Polymorphisms of mtDNA control region in Tunisian and Moroccan populations: An enrichment of forensic mtDNA databases with Northern Africa data. Forensic Science International-Genetics 3: 166-172.
15. Cherni L, Fernandes V, Pereira JB, Costa MD, Goios A, et al. (2009) Post-Last Glacial Maximum Expansion From Iberia to North Africa Revealed by Fine Characterization of mtDNA H Haplogroup in Tunisia. American Journal of Physical Anthropology 139: 253-260.
16. Watson E, Forster P, Richards M, Bandelt HJ (1997) Mitochondrial footprints of human expansions in Africa. American Journal of Human Genetics 61: 691-704.
17. Kivisild T, Reidla M, Metspalu E, Rosa A, Brehm A, et al. (2004) Ethiopian mitochondrial DNA heritage: tracking gene flow across and around the gate of tears. American Journal of Human Genetics 75: 752-770.

18. Poloni ES, Naciri Y, Bucho R, Niba R, Kervaire B, et al. (2009) Genetic Evidence for Complexity in Ethnic Differentiation and History in East Africa. Annals of Human Genetics 73: 582-600.
19. Soares P, Alshamali F, Pereira JB, Fernandes V, Silva NM, et al. (2012) The Expansion of mtDNA Haplogroup L3 within and out of Africa. Molecular Biology and Evolution 29: 915-927.
20. Non AL (2009) Mitochondrial DNA diversity of Yemenite and Ethiopian Jewish populations. American Journal of Physical Anthropology: 200-200.
21. Brandstatter A, Peterson CT, Irwin JA, Mpoke S, Koech DK, et al. (2004) Mitochondrial DNA control region sequences from Nairobi (Kenya): inferring phylogenetic parameters for the establishment of a forensic database. International Journal of Legal Medicine 118: 294-306.
22. Al-Abri A, Podgorna E, Rose JI, Pereira L, Mulligan CJ, et al. (2012) Pleistocene-Holocene boundary in Southern Arabia from the perspective of human mtDNA variation. American Journal of Physical Anthropology 149: 291-298.
23. Knight A, Underhill PA, Mortensen HM, Zhivotovsky LA, Lin AA, et al. (2003) African Y chromosome and mtDNA divergence provides insight into the history of click languages. Current Biology 13: 464-473.
24. Tishkoff SA, Gonder MK, Henn BM, Mortensen H, Knight A, et al. (2007) History of click-speaking Populations of Africa inferred from mtDNA and Y chromosome genetic variation. Molecular Biology and Evolution 24: 2180-2195.
25. Cerny V, Hajek M, Bromova M, Cmejla R, Diallo I, et al. (2006) mtDNA of Fulani nomads and their genetic relationships to neighboring sedentary populations. Human Biology 78: 9-27.
26. Quintana-Murci L, Quach H, Harmant C, Luca F, Massonnet B, et al. (2008) Maternal traces of deep common ancestry and asymmetric gene flow between Pygmy hunter-gatherers and Bantu-speaking farmers. Proc Natl Acad Sci U S A 105: 1596-1601.
27. Veeramah KR, Connell BA, Ansari Pour N, Powell A, Plaster CA, et al. (2010) Little genetic differentiation as assessed by uniparental markers in the presence of substantial language variation in peoples of the Cross River region of Nigeria. Bmc Evolutionary Biology 10: 92.
28. Mateu E, Comas D, Calafell F, Perez-Lezaun A, Abade A, et al. (1997) A tale of two islands: population history and mitochondrial DNA sequence variation of Bioko and Sao Tome, Gulf of Guinea. Annals of Human Genetics 61: 507-518.
29. Castri L, Tofanelli S, Garagnani P, Bini C, Fosella X, et al. (2009) mtDNA variability in two Bantu-speaking populations (Shona and Hutu) from Eastern Africa: implications for peopling and migration patterns in sub-Saharan Africa. American Journal of Physical Anthropology 140: 302-311.
30. Pereira L, Cerny V, Cerezo M, Silva NM, Hajek M, et al. (2010) Linking the sub-Saharan and West Eurasian gene pools: maternal and paternal heritage of the Tuareg nomads from the African Sahel. European Journal of Human Genetics 18: 915-923.
31. Jackson BA, Wilson JL, Kirbah S, Sidney SS, Rosenberger J, et al. (2005) Mitochondrial DNA genetic diversity among four ethnic groups in Sierra Leone. American Journal of Physical Anthropology 128: 156-163.
32. Trovoada MJ, Pereira L, Gusmao L, Abade A, Amorim A, et al. (2004) Pattern of mtDNA variation in three populations from Sao Tome e Principe. Annals of Human Genetics 68: 40-54.
33. Brehm A, Pereira L, Bandelt HJ, Prata MJ, Amorim A (2002) Mitochondrial portrait of the Cabo Verde archipelago: the Senegambian outpost of Atlantic slave trade. Annals of Human Genetics 66: 49-60.
34. Stefflova K, Dulik MC, Pai AA, Walker AH, Zeigler-Johnson CM, et al. (2009) Evaluation of group genetic ancestry of populations from Philadelphia and Dakar in the context of sex-biased admixture in the Americas. Plos One 4: e7842.

35. Gonzalez AM, Cabrera VM, Larruga JM, Tounkara A, Noumsi G, et al. (2006) Mitochondrial DNA variation in Mauritania and Mali and their genetic relationship to other western Africa populations. Annals of Human Genetics 70: 631-657.
36. Ely B, Wilson JL, Jackson F, Jackson BA (2006) African-American mitochondrial DNAs often match mtDNAs found in multiple African ethnic groups. Bmc Biology 4.
37. Beleza S, Gusmao L, Amorim A, Carracedo A, Salas A (2005) The genetic legacy of western Bantu migrations. Human genetics 117: 366-375.
38. Coelho M, Sequeira F, Luiselli D, Beleza S, Rocha J (2009) On the edge of Bantu expansions: mtDNA, Y chromosome and lactase persistence genetic variation in southwestern Angola. BMC evolutionary biology 9: 80.
39. Pereira L, Macaulay V, Torroni A, Scozzari R, Prata MJ, et al. (2001) Prehistoric and historic traces in the mtDNA of Mozambique: insights into the Bantu expansions and the slave trade. Annals of Human Genetics 65: 439-458.
40. Salas A, Richards M, De la Fe T, Lareu M-V, Sobrino B, et al. (2002) The making of the African mtDNA landscape. The American Journal of Human Genetics 71: 1082-1111.
41. Quintana-Murci L, Harmant C, Quach H, Balanovsky O, Zaporozhchenko V, et al. (2010) Strong maternal Khoisan contribution to the South African coloured population: a case of gender-biased admixture. The American Journal of Human Genetics 86: 611-620.
42. Chen Y-S, Olckers A, Schurr TG, Kogelnik AM, Huoponen K, et al. (2000) mtDNA variation in the South African Kung and Khwe—and their genetic relationships to other African populations. The American Journal of Human Genetics 66: 1362-1383.
43. de Filippo C, Heyn P, Barham L, Stoneking M, Pakendorf B (2010) Genetic perspectives on forager-farmer interaction in the Luangwa Valley of Zambia. American journal of physical anthropology 141: 382-394.
