# Supplementary material for: The First Modern Human Dispersals across Africa
Source: PLoS One. 2013 Nov 13;8(11):e80031. doi: 10.1371/journal.pone.0080031 (PMC3827445; doi:10.1371/journal.pone.0080031)
Supplement: Table S2 — Description of the complete mitochondrial sequences from haplogroup L0 used in this study. (PDF) [file pone.0080031.s005.pdf]

Table S2. Description of the complete mitochondrial sequences from haplogroup L0 used in this study.

**Sample Code/ Accession number**	**Region**	**Country and/or Ethnic group**	**Reference**
EU092786	Arabian Peninsula	Oman	[1]
EU092787	Arabian Peninsula	Oman	[1]
EU092925	Arabian Peninsula	Oman	[1]
EU092746	Arabian Peninsula	South Arabia	[1]
EU092745	Arabian Peninsula	South Arabia	[1]
EU092792	Arabian Peninsula	Yemen	[1]
EU092809	Arabian Peninsula	Yemen	[1]
EU092810	Arabian Peninsula	Yemen	[1]
EU092801	Arabian Peninsula	Yemen	[1]
Hide1	Central Africa	Cameroon	This study
Koto55	Central Africa	Cameroon	This study
Bula149	Central Africa	Cameroon	This study
HM771160	Central Africa	Central Africa Republic (Pygmy)	[2]
HM771161	Central Africa	Central Africa Republic (Pygmy)	[2]
Kane72	Central Africa	Chad	This study
EU092892	Central Africa	Chad	[1]
EU092881	Central Africa	Chad	[1]
EU092878	Central Africa	Chad	[1]
EU092889	Central Africa	Chad	[1]
EU092900	Central Africa	Chad	[1]
Daz39	Central Africa	Chad (Daza)	This study
Daz18	Central Africa	Chad (Daza)	This study

EU597537	Central Africa	Congo	[3]
HM771199	Central Africa	Congo (Pygmy)	[2]
AM711903	Central Africa	Congo (Pygmy)	[4]
HM771200	Central Africa	Congo (Pygmy)	[2]
HM771188	Central Africa	Congo (Pygmy)	[2]
HM771201	Central Africa	Congo (Pygmy)	[2]
HM771189	Central Africa	Congo (Pygmy)	[2]
EU597537	Central Africa	Congo (Pygmy)	[3]
HM771202	Central Africa	Congo (Pygmy)	[2]
HM771190	Central Africa	Congo (Pygmy)	[2]
AF346998	Central Africa	Congo (Pygmy)	[5]
AF346999	Central Africa	Congo (Pygmy)	[5]
AY963585	Central Africa	Ugands	[6]
EU092936	East Africa	Ethiopia	[1]
Eth14	East Africa	Ethiopia	This study
EU092945	East Africa	Ethiopia	[1]
EU092950	East Africa	Ethiopia	[1]
EU092668	East Africa	Ethiopia (Jew)	[1]
EU092670	East Africa	Ethiopia (Jew)	[1]
EF556174	East Africa	Ethiopia (Jew)	[7]
Oro33	East Africa	Ethiopia (Oromo)	This study
Oro35	East Africa	Ethiopia (Oromo)	This study
EU092909	East Africa	Kenya	[1]
EU092911	East Africa	Kenya	[1]
EU092913	East Africa	Kenya	[1]
EU092906	East Africa	Kenya	[1]

NA19027	East Africa	Kenya	[8]
NA19031	East Africa	Kenya	[8]
NA19039	East Africa	Kenya	[8]
NA19042	East Africa	Kenya	[8]
NA19311	East Africa	Kenya	[8]
NA19312	East Africa	Kenya	[8]
NA19327	East Africa	Kenya	[8]
NA19328	East Africa	Kenya	[8]
NA19350	East Africa	Kenya	[8]
NA19379	East Africa	Kenya	[8]
NA19382	East Africa	Kenya	[8]
NA19402	East Africa	Kenya	[8]
NA19430	East Africa	Kenya	[8]
NA19440	East Africa	Kenya	[8]
NA19448	East Africa	Kenya	[8]
NA19449	East Africa	Kenya	[8]
NA19454	East Africa	Kenya	[8]
NA19466	East Africa	Kenya	[8]
NA19467	East Africa	Kenya	[8]
NA19474	East Africa	Kenya	[8]
Tur15	East Africa	Kenya (Turkana)	This study
Tur5	East Africa	Kenya (Turkana)	This study
Nub85	East Africa	Nubia	This study
Nub78	East Africa	Nubia	This study
Som20	East Africa	Somalia	This study
Som58	East Africa	Somalia	This study

Som42	East Africa	Somalia	This study
Som134	East Africa	Somalia	This study
Som35	East Africa	Somalia	This study
Som92	East Africa	Somalia	This study
Som142	East Africa	Somalia	This study
Som136	East Africa	Somalia	This study
Ara2	East Africa	Sudan (Arab)	This study
Ara36	East Africa	Sudan (Arab)	This study
JQ702428	Europe	Italy	[7]
EU092665	Near East	Israel (Bedoin)	[1]
EU092760	Near East	Iran	[1]
EU092921	Near East	Kuwait	[1]
EU092819	North Africa	Algeria	[1]
EU092764	North Africa	Egypt	[1]
EU092763	North Africa	Egypt	[1]
EU935437	North Africa	Egypt	[9]
EU935464	North Africa	Egypt	[9]
EU935467	North Africa	Egypt	[9]
EU935434	North Africa	Egypt	[9]
AF381988	North Africa	Morocco	[10]
DQ341058	North America	Dominica	[11]
JQ702227	North America	Puerto Rico	[7]
HG01108	North America	Puerto Rico	[8]
EU092964	North America	USA	[1]
L127	North America	USA	[12]
DQ304903	North America	USA	[13]

DQ304901	North America	USA	[13]
DQ304902	North America	USA	[13]
DQ304904	North America	USA	[13]
L125	North America	USA	[12]
DQ304899	North America	USA	[13]
DQ304900	North America	USA	[13]
EU092963	North America	USA	[1]
L098	North America	USA	[12]
DQ304897	North America	USA	[13]
DQ304898	North America	USA	[13]
L123	North America	USA	[12]
L048	North America	USA	[12]
NA19904	North America	USA	[8]
NA20334	North America	USA	[8]
NA20336	North America	USA	[8]
EU092842	South Africa	South Africa - KhoeSan	[1]
EU092845	South Africa	South Africa - KhoeSan	[1]
EU092844	South Africa	South Africa - KhoeSan	[1]
EU092840	South Africa	South Africa - KhoeSan	[1]
EU092839	South Africa	South Africa - KhoeSan	[1]
EU092846	South Africa	South Africa - KhoeSan	[1]
AY195777	South Africa	South Africa - KhoeSan	[14]
EU092835	South Africa	South Africa - KhoeSan	[1]
EU092834	South Africa	South Africa - KhoeSan	[1]
EU092831	South Africa	South Africa - KhoeSan	[1]
AM711904	South Africa	South Africa - KhoeSan	[4]

EU092856	South Africa	South Africa - KhoeSan	[1]
EU092843	South Africa	South Africa - KhoeSan	[1]
EU092832	South Africa	South Africa - KhoeSan	[1]
EU092859	South Africa	South Africa - KhoeSan	[1]
EU092841	South Africa	South Africa - KhoeSan	[1]
EU092965	South Africa	South Africa - hoeSan	[1]
EU092833	South Africa	South Africa - KhoeSan	[1]
EU092853	South Africa	South Africa - KhoeSan	[1]
EU092855	South Africa	South Africa - KhoeSan	[1]
AF347008	South Africa	South Africa - KhoeSan	[5]
AF347009	South Africa	South Africa - KhoeSan	[5]
EU092837	South Africa	South Africa - KhoeSan	[1]
EU092858	South Africa	South Africa - KhoeSan	[1]
Moz24	South Africa	Mozambique	This study
EU092708	South Africa	Mozambique	[1]
Moz91	South Africa	Mozambique	This study
Moz236	South Africa	Mozambique	This study
EU092700	South Africa	Mozambique	[1]
Moz207	South Africa	Mozambique	This study
Moz51	South Africa	Mozambique	This study
EU092860	South Africa	Mozambique	[1]
Moz48	South Africa	Mozambique	This study
Moz74	South Africa	Mozambique	This study
Moz593	South Africa	Mozambique	This study
Moz590	South Africa	Mozambique	This study
Moz238	South Africa	Mozambique	This study

Moz31	South Africa	Mozambique	This study
EU092688	South Africa	Mozambique	[1]
Moz558	South Africa	Mozambique	This study
Moz347	South Africa	Mozambique	This study
Moz296	South Africa	Mozambique	This study
EU092701	South Africa	Mozambique	[1]
EU597502	South Africa	South Africa	[3]
EU092863	South Africa	South Africa	[1]
EU092870	South Africa	South Africa	[1]
EU092874	South Africa	South Africa	[1]
AY195780	South Africa	South Africa	[14]
EU092871	South Africa	South Africa	[1]
EU092869	South Africa	South Africa	[1]
EU092861	South Africa	South Africa	[1]
EU092868	South Africa	South Africa	[1]
KC345764	South Africa	Angola	[15]
KC345765	South Africa	Angola	[15]
KC345766	South Africa	Angola	[15]
KC345767	South Africa	Angola	[15]
KC345768	South Africa	Angola	[15]
KC345769	South Africa	Angola	[15]
KC345770	South Africa	Angola	[15]
KC345771	South Africa	Angola	[15]
KC345772	South Africa	Angola	[15]
KC345773	South Africa	Angola	[15]
KC345774	South Africa	Angola	[15]

KC345775	South Africa	Angola	[15]
KC345776	South Africa	Angola	[15]
KC345777	South Africa	Angola	[15]
KC345778	South Africa	Angola	[15]
KC345779	South Africa	Angola	[15]
KC345780	South Africa	Angola	[15]
KC345781	South Africa	Angola	[15]
KC345782	South Africa	Angola	[15]
KC345783	South Africa	Angola	[15]
KC345784	South Africa	Angola	[15]
KC345785	South Africa	Angola	[15]
KC345917	South Africa	Botswana	[15]
KC345918	South Africa	Botswana	[15]
KC345919	South Africa	Botswana	[15]
KC345920	South Africa	Botswana	[15]
KC345921	South Africa	Botswana	[15]
KC345922	South Africa	Botswana	[15]
KC345923	South Africa	Botswana	[15]
KC345924	South Africa	Botswana	[15]
KC345925	South Africa	Botswana	[15]
KC345926	South Africa	Botswana	[15]
KC345927	South Africa	Botswana	[15]
KC345928	South Africa	Botswana	[15]
KC345930	South Africa	Botswana	[15]
KC345931	South Africa	Botswana	[15]
KC345932	South Africa	Botswana	[15]

KC345933	South Africa	Botswana	[15]
KC346087	South Africa	Botswana	[15]
KC346088	South Africa	Botswana	[15]
KC346089	South Africa	Botswana	[15]
KC346090	South Africa	Botswana	[15]
KC346091	South Africa	Botswana	[15]
KC345787	South Africa	Botswana-Khoe	[15]
KC345788	South Africa	Botswana-Khoe	[15]
KC345789	South Africa	Botswana-Khoe	[15]
KC345790	South Africa	Botswana-Khoe	[15]
KC345791	South Africa	Botswana-Khoe	[15]
KC345792	South Africa	Botswana-Khoe	[15]
KC345793	South Africa	Botswana-Khoe	[15]
KC345794	South Africa	Botswana-Khoe	[15]
KC345795	South Africa	Botswana-Khoe	[15]
KC345796	South Africa	Botswana-Khoe	[15]
KC345797	South Africa	Botswana-Khoe	[15]
KC345798	South Africa	Botswana-Khoe	[15]
KC345799	South Africa	Botswana-Khoe	[15]
KC345800	South Africa	Botswana-Khoe	[15]
KC345801	South Africa	Botswana-Khoe	[15]
KC345802	South Africa	Botswana-Khoe	[15]
KC345803	South Africa	Botswana-Khoe	[15]
KC345804	South Africa	Botswana-Khoe	[15]
KC345805	South Africa	Botswana-Khoe	[15]
KC345806	South Africa	Botswana-Khoe	[15]

KC345807	South Africa	Botswana-Khoe	[15]
KC345808	South Africa	Botswana-Khoe	[15]
KC345810	South Africa	Botswana-Khoe	[15]
KC345811	South Africa	Botswana-Khoe	[15]
KC345812	South Africa	Botswana-Khoe	[15]
KC345813	South Africa	Botswana-Khoe	[15]
KC345814	South Africa	Botswana-Khoe	[15]
KC345815	South Africa	Botswana-Khoe	[15]
KC345816	South Africa	Botswana-Khoe	[15]
KC345817	South Africa	Botswana-Khoe	[15]
KC345818	South Africa	Botswana-Khoe	[15]
KC345819	South Africa	Botswana-Khoe	[15]
KC345820	South Africa	Botswana-Khoe	[15]
KC345821	South Africa	Botswana-Khoe	[15]
KC345822	South Africa	Botswana-Khoe	[15]
KC345823	South Africa	Botswana-Khoe	[15]
KC345824	South Africa	Botswana-Khoe	[15]
KC345825	South Africa	Botswana-Khoe	[15]
KC345826	South Africa	Botswana-Khoe	[15]
KC345827	South Africa	Botswana-Khoe	[15]
KC345828	South Africa	Botswana-Khoe	[15]
KC345830	South Africa	Botswana-Khoe	[15]
KC345831	South Africa	Botswana-Khoe	[15]
KC345832	South Africa	Botswana-Khoe	[15]
KC345833	South Africa	Botswana-Khoe	[15]
KC345834	South Africa	Botswana-Khoe	[15]

KC345835	South Africa	Botswana-Khoe	[15]
KC345836	South Africa	Botswana-Khoe	[15]
KC345837	South Africa	Botswana-Khoe	[15]
KC345838	South Africa	Botswana-Khoe	[15]
KC345839	South Africa	Botswana-Khoe	[15]
KC345840	South Africa	Botswana-Khoe	[15]
KC345841	South Africa	Botswana-Khoe	[15]
KC345842	South Africa	Botswana-Khoe	[15]
KC345843	South Africa	Botswana-Khoe	[15]
KC345844	South Africa	Botswana-Khoe	[15]
KC345845	South Africa	Botswana-Khoe	[15]
KC345846	South Africa	Botswana-Khoe	[15]
KC345847	South Africa	Botswana-Khoe	[15]
KC345848	South Africa	Botswana-Khoe	[15]
KC345849	South Africa	Botswana-Khoe	[15]
KC345850	South Africa	Botswana-Khoe	[15]
KC345851	South Africa	Botswana-Khoe	[15]
KC345934	South Africa	Botswana-Khoe	[15]
KC345935	South Africa	Botswana-Khoe	[15]
KC345936	South Africa	Botswana-Khoe	[15]
KC345937	South Africa	Botswana-Khoe	[15]
KC345938	South Africa	Botswana-Khoe	[15]
KC345939	South Africa	Botswana-Khoe	[15]
KC345940	South Africa	Botswana-Khoe	[15]
KC345941	South Africa	Botswana-Khoe	[15]
KC345942	South Africa	Botswana-Khoe	[15]

KC345943	South Africa	Botswana-Khoe	[15]
KC345944	South Africa	Botswana-Khoe	[15]
KC345945	South Africa	Botswana-Khoe	[15]
KC345946	South Africa	Botswana-Khoe	[15]
KC345947	South Africa	Botswana-Khoe	[15]
KC345948	South Africa	Botswana-Khoe	[15]
KC345949	South Africa	Botswana-Khoe	[15]
KC345950	South Africa	Botswana-Khoe	[15]
KC345951	South Africa	Botswana-Khoe	[15]
KC345952	South Africa	Botswana-Khoe	[15]
KC345953	South Africa	Botswana-Khoe	[15]
KC345954	South Africa	Botswana-Khoe	[15]
KC345955	South Africa	Botswana-Khoe	[15]
KC345956	South Africa	Botswana-Khoe	[15]
KC345957	South Africa	Botswana-Khoe	[15]
KC345959	South Africa	Botswana-Khoe	[15]
KC345960	South Africa	Botswana-Khoe	[15]
KC345961	South Africa	Botswana-Khoe	[15]
KC345963	South Africa	Botswana-Khoe	[15]
KC345964	South Africa	Botswana-Khoe	[15]
KC345965	South Africa	Botswana-Khoe	[15]
KC345966	South Africa	Botswana-Khoe	[15]
KC345967	South Africa	Botswana-Khoe	[15]
KC345968	South Africa	Botswana-Khoe	[15]
KC345969	South Africa	Botswana-Khoe	[15]
KC345970	South Africa	Botswana-Khoe	[15]

KC345971	South Africa	Botswana-Khoe	[15]
KC345972	South Africa	Botswana-Khoe	[15]
KC345973	South Africa	Botswana-Khoe	[15]
KC345974	South Africa	Botswana-Khoe	[15]
KC345975	South Africa	Botswana-Khoe	[15]
KC345976	South Africa	Botswana-Khoe	[15]
KC345977	South Africa	Botswana-Khoe	[15]
KC345978	South Africa	Botswana-Khoe	[15]
KC345979	South Africa	Botswana-Khoe	[15]
KC345980	South Africa	Botswana-Khoe	[15]
KC345981	South Africa	Botswana-Khoe	[15]
KC345982	South Africa	Botswana-Khoe	[15]
KC345995	South Africa	Botswana-Khoe	[15]
KC346066	South Africa	Botswana-Khoe	[15]
KC346067	South Africa	Botswana-Khoe	[15]
KC346068	South Africa	Botswana-Khoe	[15]
KC346069	South Africa	Botswana-Khoe	[15]
KC346070	South Africa	Botswana-Khoe	[15]
KC346071	South Africa	Botswana-Khoe	[15]
KC346072	South Africa	Botswana-Khoe	[15]
KC346073	South Africa	Botswana-Khoe	[15]
KC346074	South Africa	Botswana-Khoe	[15]
KC346075	South Africa	Botswana-Khoe	[15]
KC346076	South Africa	Botswana-Khoe	[15]
KC346078	South Africa	Botswana-Khoe	[15]
KC346079	South Africa	Botswana-Khoe	[15]

KC346080	South Africa	Botswana-Khoe	[15]
KC346081	South Africa	Botswana-Khoe	[15]
KC346082	South Africa	Botswana-Khoe	[15]
KC346083	South Africa	Botswana-Khoe	[15]
KC346084	South Africa	Botswana-Khoe	[15]
KC346085	South Africa	Botswana-Khoe	[15]
KC346086	South Africa	Botswana-Khoe	[15]
KC345809	South Africa	Botswana-Kx'a	[15]
KC345852	South Africa	Botswana-Kx'a	[15]
KC345853	South Africa	Botswana-Kx'a	[15]
KC345854	South Africa	Botswana-Kx'a	[15]
KC345855	South Africa	Botswana-Kx'a	[15]
KC345856	South Africa	Botswana-Kx'a	[15]
KC345857	South Africa	Botswana-Kx'a	[15]
KC345858	South Africa	Botswana-Kx'a	[15]
KC345859	South Africa	Botswana-Kx'a	[15]
KC345860	South Africa	Botswana-Kx'a	[15]
KC345861	South Africa	Botswana-Kx'a	[15]
KC345862	South Africa	Botswana-Kx'a	[15]
KC345863	South Africa	Botswana-Kx'a	[15]
KC345864	South Africa	Botswana-Kx'a	[15]
KC345865	South Africa	Botswana-Kx'a	[15]
KC345866	South Africa	Botswana-Kx'a	[15]
KC345867	South Africa	Botswana-Kx'a	[15]
KC345868	South Africa	Botswana-Kx'a	[15]
KC345869	South Africa	Botswana-Kx'a	[15]

KC345870	South Africa	Botswana-Kx'a	[15]
KC345871	South Africa	Botswana-Kx'a	[15]
KC345872	South Africa	Botswana-Kx'a	[15]
KC345873	South Africa	Botswana-Kx'a	[15]
KC345874	South Africa	Botswana-Kx'a	[15]
KC345875	South Africa	Botswana-Kx'a	[15]
KC345876	South Africa	Botswana-Kx'a	[15]
KC345877	South Africa	Botswana-Kx'a	[15]
KC345878	South Africa	Botswana-Kx'a	[15]
KC345879	South Africa	Botswana-Kx'a	[15]
KC345880	South Africa	Botswana-Kx'a	[15]
KC345881	South Africa	Botswana-Kx'a	[15]
KC345882	South Africa	Botswana-Kx'a	[15]
KC345883	South Africa	Botswana-Kx'a	[15]
KC345884	South Africa	Botswana-Kx'a	[15]
KC345885	South Africa	Botswana-Kx'a	[15]
KC345886	South Africa	Botswana-Kx'a	[15]
KC345887	South Africa	Botswana-Kx'a	[15]
KC345888	South Africa	Botswana-Kx'a	[15]
KC345889	South Africa	Botswana-Kx'a	[15]
KC345890	South Africa	Botswana-Kx'a	[15]
KC345891	South Africa	Botswana-Kx'a	[15]
KC345892	South Africa	Botswana-Kx'a	[15]
KC345893	South Africa	Botswana-Kx'a	[15]
KC345894	South Africa	Botswana-Kx'a	[15]
KC345895	South Africa	Botswana-Kx'a	[15]

KC345896	South Africa	Botswana-Kx'a	[15]
KC345897	South Africa	Botswana-Kx'a	[15]
KC345898	South Africa	Botswana-Kx'a	[15]
KC345899	South Africa	Botswana-Kx'a	[15]
KC345900	South Africa	Botswana-Kx'a	[15]
KC345901	South Africa	Botswana-Kx'a	[15]
KC345902	South Africa	Botswana-Kx'a	[15]
KC345903	South Africa	Botswana-Kx'a	[15]
KC345904	South Africa	Botswana-Kx'a	[15]
KC345905	South Africa	Botswana-Kx'a	[15]
KC345906	South Africa	Botswana-Kx'a	[15]
KC345907	South Africa	Botswana-Kx'a	[15]
KC345908	South Africa	Botswana-Kx'a	[15]
KC345909	South Africa	Botswana-Kx'a	[15]
KC345910	South Africa	Botswana-Kx'a	[15]
KC345911	South Africa	Botswana-Kx'a	[15]
KC345912	South Africa	Botswana-Kx'a	[15]
KC345914	South Africa	Botswana-Kx'a	[15]
KC345915	South Africa	Botswana-Kx'a	[15]
KC345916	South Africa	Botswana-Kx'a	[15]
KC345983	South Africa	Botswana-Tuu	[15]
KC345984	South Africa	Botswana-Tuu	[15]
KC345985	South Africa	Botswana-Tuu	[15]
KC345986	South Africa	Botswana-Tuu	[15]
KC345987	South Africa	Botswana-Tuu	[15]
KC345988	South Africa	Botswana-Tuu	[15]

KC345989	South Africa	Botswana-Tuu	[15]
KC345990	South Africa	Botswana-Tuu	[15]
KC345991	South Africa	Botswana-Tuu	[15]
KC345992	South Africa	Botswana-Tuu	[15]
KC345993	South Africa	Botswana-Tuu	[15]
KC345994	South Africa	Botswana-Tuu	[15]
KC345996	South Africa	Botswana-Tuu	[15]
KC345997	South Africa	Botswana-Tuu	[15]
KC345998	South Africa	Botswana-Tuu	[15]
KC345999	South Africa	Botswana-Tuu	[15]
KC346000	South Africa	Botswana-Tuu	[15]
KC346001	South Africa	Botswana-Tuu	[15]
KC346002	South Africa	Botswana-Tuu	[15]
KC346003	South Africa	Botswana-Tuu	[15]
KC346004	South Africa	Botswana-Tuu	[15]
KC346005	South Africa	Botswana-Tuu	[15]
KC346006	South Africa	Botswana-Tuu	[15]
KC346007	South Africa	Botswana-Tuu	[15]
KC346008	South Africa	Botswana-Tuu	[15]
KC346009	South Africa	Botswana-Tuu	[15]
KC346010	South Africa	Botswana-Tuu	[15]
KC346011	South Africa	Botswana-Tuu	[15]
KC346012	South Africa	Botswana-Tuu	[15]
KC346013	South Africa	Botswana-Tuu	[15]
KC346014	South Africa	Botswana-Tuu	[15]
KC346015	South Africa	Botswana-Tuu	[15]

KC346016	South Africa	Botswana-Tuu	[15]
KC346017	South Africa	Botswana-Tuu	[15]
KC346018	South Africa	Botswana-Tuu	[15]
KC346019	South Africa	Botswana-Tuu	[15]
KC346020	South Africa	Botswana-Tuu	[15]
KC346021	South Africa	Botswana-Tuu	[15]
KC346022	South Africa	Botswana-Tuu	[15]
KC346023	South Africa	Botswana-Tuu	[15]
KC346024	South Africa	Botswana-Tuu	[15]
KC346025	South Africa	Botswana-Tuu	[15]
KC346026	South Africa	Botswana-Tuu	[15]
KC346027	South Africa	Botswana-Tuu	[15]
KC346028	South Africa	Botswana-Tuu	[15]
KC346029	South Africa	Botswana-Tuu	[15]
KC346030	South Africa	Botswana-Tuu	[15]
KC346031	South Africa	Botswana-Tuu	[15]
KC346032	South Africa	Botswana-Tuu	[15]
KC346033	South Africa	Botswana-Tuu	[15]
KC346034	South Africa	Botswana-Tuu	[15]
KC346035	South Africa	Botswana-Tuu	[15]
KC346036	South Africa	Botswana-Tuu	[15]
KC346037	South Africa	Botswana-Tuu	[15]
KC346038	South Africa	Botswana-Tuu	[15]
KC346039	South Africa	Botswana-Tuu	[15]
KC346040	South Africa	Botswana-Tuu	[15]
KC346041	South Africa	Botswana-Tuu	[15]

KC346042	South Africa	Botswana-Tuu	[15]
KC346043	South Africa	Botswana-Tuu	[15]
KC346044	South Africa	Botswana-Tuu	[15]
KC346045	South Africa	Botswana-Tuu	[15]
KC346046	South Africa	Botswana-Tuu	[15]
KC346047	South Africa	Botswana-Tuu	[15]
KC346048	South Africa	Botswana-Tuu	[15]
KC346049	South Africa	Botswana-Tuu	[15]
KC346050	South Africa	Botswana-Tuu	[15]
KC346051	South Africa	Botswana-Tuu	[15]
KC346052	South Africa	Botswana-Tuu	[15]
KC346053	South Africa	Botswana-Tuu	[15]
KC346054	South Africa	Botswana-Tuu	[15]
KC346055	South Africa	Botswana-Tuu	[15]
KC346056	South Africa	Botswana-Tuu	[15]
KC346057	South Africa	Botswana-Tuu	[15]
KC346058	South Africa	Botswana-Tuu	[15]
KC346059	South Africa	Botswana-Tuu	[15]
KC346060	South Africa	Botswana-Tuu	[15]
KC346061	South Africa	Botswana-Tuu	[15]
KC346062	South Africa	Botswana-Tuu	[15]
KC346063	South Africa	Botswana-Tuu	[15]
KC346064	South Africa	Botswana-Tuu	[15]
KC346065	South Africa	Botswana-Tuu	[15]
KC345958	South Africa	Namibia-Khoe	[15]
KC346092	South Africa	Namibia-Khoe	[15]

KC346093	South Africa	Namibia - Khoe	[15]
KC346094	South Africa	Namibia - Khoe	[15]
KC346095	South Africa	Namibia - Khoe	[15]
KC346096	South Africa	Namibia - Khoe	[15]
KC346097	South Africa	Namibia - Khoe	[15]
KC346098	South Africa	Namibia - Khoe	[15]
KC346099	South Africa	Namibia - Khoe	[15]
KC346100	South Africa	Namibia - Khoe	[15]
KC346101	South Africa	Namibia - Khoe	[15]
KC346102	South Africa	Namibia - Khoe	[15]
KC346103	South Africa	Namibia - Khoe	[15]
KC346104	South Africa	Namibia - Khoe	[15]
KC346105	South Africa	Namibia - Khoe	[15]
KC346106	South Africa	Namibia - Khoe	[15]
KC346107	South Africa	Namibia - Khoe	[15]
KC346108	South Africa	Namibia - Khoe	[15]
KC346109	South Africa	Namibia - Khoe	[15]
KC346110	South Africa	Namibia - Khoe	[15]
KC346111	South Africa	Namibia - Khoe	[15]
KC346112	South Africa	Namibia - Khoe	[15]
KC346113	South Africa	Namibia - Khoe	[15]
KC346114	South Africa	Namibia - Khoe	[15]
KC346115	South Africa	Namibia - Khoe	[15]
KC346116	South Africa	Namibia - Khoe	[15]
KC346117	South Africa	Namibia - Khoe	[15]
KC346118	South Africa	Namibia - Khoe	[15]

KC346119	South Africa	Namibia - Khoe	[15]
KC346120	South Africa	Namibia - Khoe	[15]
KC346121	South Africa	Namibia - Khoe	[15]
KC346122	South Africa	Namibia - Khoe	[15]
KC346123	South Africa	Namibia - Khoe	[15]
KC346124	South Africa	Namibia - Khoe	[15]
KC346125	South Africa	Namibia - Khoe	[15]
KC346126	South Africa	Namibia - Khoe	[15]
KC346127	South Africa	Namibia - Khoe	[15]
KC346128	South Africa	Namibia - Khoe	[15]
KC346129	South Africa	Namibia - Khoe	[15]
KC346130	South Africa	Namibia - Khoe	[15]
KC346131	South Africa	Namibia - Khoe	[15]
KC346132	South Africa	Namibia - Khoe	[15]
KC346133	South Africa	Namibia - Khoe	[15]
KC346134	South Africa	Namibia - Khoe	[15]
KC346135	South Africa	Namibia - Khoe	[15]
KC346136	South Africa	Namibia - Khoe	[15]
KC346137	South Africa	Namibia - Khoe	[15]
KC346138	South Africa	Namibia - Khoe	[15]
KC346139	South Africa	Namibia - Khoe	[15]
KC346140	South Africa	Namibia - Khoe	[15]
KC346141	South Africa	Namibia - Khoe	[15]
KC346142	South Africa	Namibia - Khoe	[15]
KC346143	South Africa	Namibia - Khoe	[15]
KC346144	South Africa	Namibia - Khoe	[15]

KC346145	South Africa	Namibia - Khoe	[15]
KC346146	South Africa	Namibia - Khoe	[15]
KC346147	South Africa	Namibia - Khoe	[15]
KC346148	South Africa	Namibia - Khoe	[15]
KC346149	South Africa	Namibia - Khoe	[15]
KC346150	South Africa	Namibia - Khoe	[15]
KC346151	South Africa	Namibia - Khoe	[15]
KC346152	South Africa	Namibia - Khoe	[15]
KC346153	South Africa	Namibia - Khoe	[15]
KC346154	South Africa	Namibia - Khoe	[15]
KC346155	South Africa	Namibia - Khoe	[15]
KC346156	South Africa	Namibia - Khoe	[15]
KC346157	South Africa	Namibia - Khoe	[15]
KC346158	South Africa	Namibia - Khoe	[15]
KC346159	South Africa	Namibia - Khoe	[15]
KC346160	South Africa	Namibia - Khoe	[15]
KC346161	South Africa	Namibia - Khoe	[15]
KC346162	South Africa	Namibia - Khoe	[15]
KC346163	South Africa	Namibia - Khoe	[15]
KC346164	South Africa	Namibia - Khoe	[15]
KC346165	South Africa	Namibia - Khoe	[15]
KC346166	South Africa	Namibia - Khoe	[15]
KC346167	South Africa	Namibia - Khoe	[15]
KC346168	South Africa	Namibia - Khoe	[15]
KC346169	South Africa	Namibia - Khoe	[15]
KC346170	South Africa	Namibia - Khoe	[15]

KC346171	South Africa	Namibia - Khoe	[15]
KC346172	South Africa	Namibia - Khoe	[15]
KC346173	South Africa	Namibia - Khoe	[15]
KC346174	South Africa	Namibia - Khoe	[15]
KC346175	South Africa	Namibia - Khoe	[15]
KC346176	South Africa	Namibia - Khoe	[15]
KC346177	South Africa	Namibia - Khoe	[15]
KC346178	South Africa	Namibia - Khoe	[15]
KC346179	South Africa	Namibia - Khoe	[15]
KC346180	South Africa	Namibia - Khoe	[15]
KC346181	South Africa	Namibia - Khoe	[15]
KC346182	South Africa	Namibia - Khoe	[15]
KC346183	South Africa	Namibia - Khoe	[15]
KC346184	South Africa	Namibia - Khoe	[15]
KC346185	South Africa	Namibia - Khoe	[15]
KC346186	South Africa	Namibia - Khoe	[15]
KC346187	South Africa	Namibia - Khoe	[15]
KC346188	South Africa	Namibia - Khoe	[15]
KC346189	South Africa	Namibia - Khoe	[15]
KC346190	South Africa	Namibia - Khoe	[15]
KC346191	South Africa	Namibia - Khoe	[15]
KC346192	South Africa	Namibia - Khoe	[15]
KC346193	South Africa	Namibia - Khoe	[15]
KC346194	South Africa	Namibia - Khoe	[15]
KC346195	South Africa	Namibia - Khoe	[15]
KC346196	South Africa	Namibia - Khoe	[15]

KC346197	South Africa	Namibia - Khoe	[15]
KC346198	South Africa	Namibia - Khoe	[15]
KC346199	South Africa	Namibia - Khoe	[15]
KC346200	South Africa	Namibia - Khoe	[15]
KC346201	South Africa	Namibia - Khoe	[15]
KC346202	South Africa	Namibia - Khoe	[15]
KC346203	South Africa	Namibia - Khoe	[15]
KC346204	South Africa	Namibia - Khoe	[15]
KC346205	South Africa	Namibia - Khoe	[15]
KC346206	South Africa	Namibia - Khoe	[15]
KC346207	South Africa	Namibia - Khoe	[15]
KC346208	South Africa	Namibia - Khoe	[15]
KC346209	South Africa	Namibia - Khoe	[15]
KC346210	South Africa	Namibia - Khoe	[15]
KC346211	South Africa	Namibia - Khoe	[15]
KC346212	South Africa	Namibia - Khoe	[15]
KC346213	South Africa	Namibia - Khoe	[15]
KC346214	South Africa	Namibia - Khoe	[15]
KC346215	South Africa	Namibia - Khoe	[15]
KC346216	South Africa	Namibia - Khoe	[15]
KC346217	South Africa	Namibia - Khoe	[15]
KC346218	South Africa	Namibia - Khoe	[15]
KC346219	South Africa	Namibia - Khoe	[15]
KC346220	South Africa	Namibia - Khoe	[15]
KC346221	South Africa	Namibia - Khoe	[15]
KC346222	South Africa	Namibia - Khoe	[15]

KC346223	South Africa	Namibia - Khoe	[15]
KC346224	South Africa	Namibia - Khoe	[15]
KC346225	South Africa	Namibia - Khoe	[15]
KC346226	South Africa	Namibia - Khoe	[15]
KC346227	South Africa	Namibia - Khoe	[15]
KC346228	South Africa	Namibia - Khoe	[15]
KC346229	South Africa	Namibia - Khoe	[15]
KC346230	South Africa	Namibia - Khoe	[15]
KC346231	South Africa	Namibia - Khoe	[15]
KC346232	South Africa	Namibia - Khoe	[15]
JX303745	South Africa	Zambia	[16]
JX303753	South Africa	Zambia	[16]
JX303757	South Africa	Zambia	[16]
JX303762	South Africa	Zambia	[16]
JX303763	South Africa	Zambia	[16]
JX303765	South Africa	Zambia	[16]
JX303766	South Africa	Zambia	[16]
JX303772	South Africa	Zambia	[16]
JX303778	South Africa	Zambia	[16]
JX303784	South Africa	Zambia	[16]
JX303786	South Africa	Zambia	[16]
JX303788	South Africa	Zambia	[16]
JX303791	South Africa	Zambia	[16]
JX303796	South Africa	Zambia	[16]
JX303817	South Africa	Zambia	[16]
JX303818	South Africa	Zambia	[16]

JX303823	South Africa	Zambia	[16]
JX303826	South Africa	Zambia	[16]
JX303830	South Africa	Zambia	[16]
JX303831	South Africa	Zambia	[16]
JX303835	South Africa	Zambia	[16]
JX303856	South Africa	Zambia	[16]
JX303861	South Africa	Zambia	[16]
JX303865	South Africa	Zambia	[16]
JX303867	South Africa	Zambia	[16]
JX303868	South Africa	Zambia	[16]
JX303869	South Africa	Zambia	[16]
JX303895	South Africa	Zambia	[16]
JX303903	South Africa	Zambia	[16]
JX303904	South Africa	Zambia	[16]
JX303911	South Africa	Zambia	[16]
KC346233	South Africa	Zambia	[15]
KC346234	South Africa	Zambia	[15]
KC346235	South Africa	Zambia	[15]
KC346236	South Africa	Zambia	[15]
KC346237	South Africa	Zambia	[15]
KC346238	South Africa	Zambia	[15]
KC346239	South Africa	Zambia	[15]
KC346240	South Africa	Zambia	[15]
KC346241	South Africa	Zambia	[15]
KC346242	South Africa	Zambia	[15]
KC346243	South Africa	Zambia	[15]

KC346244	South Africa	Zambia	[15]
KC346245	South Africa	Zambia	[15]
KC346246	South Africa	Zambia	[15]
KC346247	South Africa	Zambia	[15]
KC346248	South Africa	Zambia	[15]
FJ157838	South Asia	India	[17]
FJ157839	South Asia	India	[17]
FJ157840	South Asia	India	[17]
AF346985	Unknown	Hausa individual	[5]
JQ703481	Unknown	Unknown	[7]
JQ702326	Unknown	Unknown	[7]
JQ705109	Unknown	Unknown	[7]
JQ044903	West Africa	Burkina Faso	[18]
JQ044851	West Africa	Burkina Faso	[18]
JQ044893	West Africa	Burkina Faso	[18]
JQ044943	West Africa	Burkina Faso	[18]
JQ045053	West Africa	Burkina Faso	[18]
JQ044874	West Africa	Burkina Faso	[18]
JQ044838	West Africa	Burkina Faso	[18]
JQ044849	West Africa	Burkina Faso	[18]
JQ045004	West Africa	Burkina Faso	[18]
JQ044995	West Africa	Burkina Faso	[18]
NA18510	West Africa	Yoruba	[8]
NA18861	West Africa	Yoruba	[8]
NA18876	West Africa	Yoruba	[8]
NA18877	West Africa	Yoruba	[8]

NA19137	West Africa	Yoruba	[8]
NA19156	West Africa	Yoruba	[8]
NA19216	West Africa	Yoruba	[8]
EU092714	West Africa	Guiné Bissau	[1]
FZin27	West Africa	Niger	This study
STP168	West Africa	São Tomé and Príncipe	This study
STP117	West Africa	São Tomé and Príncipe	This study
STP26	West Africa	São Tomé and Príncipe	This study
STP1	West Africa	São Tomé and Príncipe	This study

References

1. Behar DM, Villems R, Soodyall H, Blue-Smith J, Pereira L, et al. (2008) The dawn of human matrilineal diversity. American Journal of Human Genetics 82: 1130-1140.
2. Batini C, Lopes J, Behar DM, Calafell F, Jorde LB, et al. (2011) Insights into the Demographic History of African Pygmies from Complete Mitochondrial Genomes. Molecular Biology and Evolution 28: 1099-1110.
3. Hartmann A, Thieme M, Nanduri LK, Stempfl T, Moehle C, et al. (2009) Validation of microarray-based resequencing of 93 worldwide mitochondrial genomes. Hum Mutat 30: 115-122.
4. Arnason U, Gullberg A, Janke A, Kullberg M (2007) Mitogenomic analyses of caniform relationships. Molecular Phylogenetics and Evolution 45: 863-874.
5. Ingman M, Kaessmann H, Paabo S, Gyllensten U (2000) Mitochondrial genome variation and the origin of modern humans. Nature 408: 708-713.
6. Macaulay V, Hill C, Achilli A, Rengo C, Clarke D, et al. (2005) Single, rapid coastal settlement of Asia revealed by analysis of complete mitochondrial genomes. Science 308: 1034-1036.
7. Behar DM, van Oven M, Rosset S, Metspalu M, Loogvali EL, et al. (2012) A "Copernican" Reassessment of the Human Mitochondrial DNA Tree from its Root. American Journal of Human Genetics 90: 675-684.
8. Zheng H-X, Yan S, Qin Z-D, Jin L (2012) MtDNA analysis of global populations support that major population expansions began before Neolithic Time. Scientific reports 2.
9. Kujanova M, Pereira L, Fernandes V, Pereira JB, Cerny V (2009) Near eastern neolithic genetic input in a small oasis of the Egyptian Western Desert. American Journal of Physical Anthropology 140: 336-346.
10. Maca-Meyer N, Gonzalez AM, Larruga JM, Flores C, Cabrera VM (2001) Major genomic mitochondrial lineages delineate early human expansions. Bmc Genetics 2: 13.
11. Torroni A, Achilli A, Macaulay V, Richards M, Bandelt HJ (2006) Harvesting the fruit of the human mtDNA tree. Trends Genet 22: 339-345.

12. Howell N, Elson JL, Howell C, Turnbull DM (2007) Relative rates of evolution in the coding and control regions of African mtDNAs. Molecular Biology and Evolution 24: 2213-2221.
13. Just RS, Diegoli TM, Saunier JL, Irwin JA, Parsons TJ (2008) Complete mitochondrial genome sequences for 265 African American and U.S. "Hispanic" individuals. Forensic Sci Int Genet 2: e45-48.
14. Mishmar D, Ruiz-Pesini E, Golik P, Macaulay V, Clark AG, et al. (2003) Natural selection shaped regional mtDNA variation in humans. Proc Natl Acad Sci U S A 100: 171-176.
15. Barbieri C, Vicente M, Rocha J, Mpoloka SW, Stoneking M, et al. (2013) Ancient Substructure in Early mtDNA Lineages of Southern Africa. The American Journal of Human Genetics 92: 285-292.
16. Barbieri C, Butthof A, Bostoen K, Pakendorf B (2012) Genetic perspectives on the origin of clicks in Bantu languages from southwestern Zambia. European Journal of Human Genetics.
17. Eaaswarkhanth M, Haque I, Ravesh Z, Romero IG, Meganathan PR, et al. (2010) Traces of sub-Saharan and Middle Eastern lineages in Indian Muslim populations. European Journal of Human Genetics 18: 354-363.
18. Barbieri C, Whitten M, Beyer K, Schreiber H, Li M, et al. (2012) Contrasting maternal and paternal histories in the linguistic context of Burkina Faso. Molecular Biology and Evolution 29: 1213-1223.
