# Supplementary material for: The First Modern Human Dispersals across Africa
Source: PLoS One. 2013 Nov 13;8(11):e80031. doi: 10.1371/journal.pone.0080031 (PMC3827445; doi:10.1371/journal.pone.0080031)
Supplement: Table S3 — 224 random sequences used in an overall African mtDNA tree. (PDF) [file pone.0080031.s006.pdf]

Table S3. 224 random sequences used in an overall African mtDNA tree.

Tur15*	DQ341075	EU092786	EU092943	JN655778	JQ044936
Moz558*	DQ341076	EU092787	EU092944	JN655780	JQ044939
Som20*	DQ341078	EU092792	EU092949	JN655784	JQ044941
Som142*	DQ341080	EU092802	EU092964	JN655785	JQ044959
Som136*	DQ341081	EU092813	EU092965	JN655786	JQ044975
Som58*	EF556173	EU092817	EU273489	JN655787	JQ045002
Bula149*	EU092660	EU092818	EU273491	JN655788	JQ045008
EU092936	EU092661	EU092822	EU273493	JN655794	JQ045029
HM771117	EU092671	EU092824	EU273499	JN655797	JQ045030
DQ341061	EU092676	EU092831	EU273501	JN655798	JQ045038
JN655776	EU092678	EU092832	EU597500	JN655803	JQ045043
EU273484	EU092686	EU092833	EU597502	JN655812	JQ045062
DQ282507	EU092693	EU092837	EU597570	JN655813	JQ045070
AF346987	EU092695	EU092838	EU935440	JN655815	JQ045074
AF346995	EU092697	EU092840	FJ460520	JN655825	JQ045080
AF346999	EU092698	EU092848	FJ460531	JN655830	JQ045084
AY195785	EU092700	EU092851	FJ625848	JN655837	JQ045092
AY963585	EU092703	EU092853	FJ625856	JQ044795	JQ045101
DQ304924	EU092708	EU092863	GU455418	JQ044797	JQ045111
DQ304925	EU092709	EU092870	HM596745	JQ044810	JQ701901
DQ304926	EU092712	EU092878	HM771114	JQ044811	JQ701954
EU092845	EU092715	EU092885	HM771136	JQ044816	JQ702441
EU092770	EU092717	EU092886	HM771162	JQ044829	JQ702617
EU092877	EU092724	EU092888	HM771166	JQ044831	JQ702626
EU092699	EU092734	EU092890	HM771171	JQ044834	JQ702659
DQ304928	EU092736	EU092891	HM771178	JQ044836	JQ703773
DQ304945	EU092740	EU092898	HM771184	JQ044843	JQ703986
DQ304946	EU092747	EU092902	HM771203	JQ044846	JQ704919
DQ304949	EU092748	EU092906	HM771204	JQ044847	JQ705275
DQ304954	EU092750	EU092913	HM771206	JQ044858	JQ705310
DQ304985	EU092752	EU092915	HM771211	JQ044866	JQ705521
DQ305010	EU092766	EU092916	HM771220	JQ044871	JQ705626
DQ305018	EU092768	EU092921	HM771223	JQ044878	JQ705650
DQ341063	EU092773	EU092923	HM771226	JQ044882	JQ045026
DQ341064	EU092774	EU092934	HM771233	JQ044907	
DQ341065	EU092776	EU092935	HQ425328	JQ044910	
DQ341069	EU092781	EU092941	HQ425645	JQ044914	
DQ341074	EU092784	EU092942	JN655774	JQ044922	

*This study
